# Together4RD position statement on collaboration between European reference networks and industry

**DOI:** 10.1186/s13023-023-02853-9

**Published:** 2023-09-05

**Authors:** Victoria Hedley, Matt Bolz-Johnson, Ines Hernando, Rosalind Kenward, Rima Nabbout, Clara Romero, Franz Schaefer, Sheela Upadhyaya, Together4RD Steering Group, Alexis Arzimanoglou, Hélène Dollfus, Dorothée Leroux, Maurizio Scarpa, Franz Schaefer, Alain Verloes, Matt Bolz-Johnson, Daria Julkowska, Ana Rath, Victoria Hedley, Anton Ussi, Yanis Mimouni, Rima Nabbout, Morgane Cuisenier, Anne-Sophie Chalandon, Toon Digneffe, Gabriella Almberg, Matteo Scarabelli, Leander Vranken

**Affiliations:** 1 Newcastle University, Newcastle Upon Tyne, UK; 2 EURORDIS (Rare Diseases Europe), Paris, France; 3 FIPRA International, Brussels, Belgium; 4 Pediatric Neurology Department, Hôpital Necker Enfants Malades, APHP, Universite Paris Cité, Institut Imagine, Paris, France; 5 Center For Pediatrics and Adolescent Medicine, University of Heidelberg, Heidelberg, Germany

**Keywords:** Rare disease, European reference network, Public–private-partnership, Rare diseases, ERNs, Networking, Research

## Abstract

Notwithstanding two decades of policy and legislation in Europe, aimed to foster research and development in rare conditions, only 5–6% of rare diseases have dedicated treatments. Given with the huge number of conditions classed as rare (which is increasing all the time), this equates to major unmet need for patients (over 30 million in the EU alone). Worryingly, the pace of Research and Innovation in Europe is lagging behind other regions of the world, and a seismic shift in the way in which research is planned and delivered is required, in order to remain competitive and—most importantly—bring meaningful, disease-altering treatments to those who desperately need them. The European Reference Networks (ERNs), launched in 2017, hold major potential to alleviate many of these challenges, and more, but only if adequately supported (financially, technically, and via robust policies and infrastructure) to *realise* that potential: and even then, only if able to forge robust collaborations harnessing the expertise, resources, knowledge and data of all stakeholders involved in rare disease, including Industry. To-date, however, ERN-Industry interactions have been largely limited, for a range of reasons (concerning barriers both tangible and perceived). This Position Statement analyses these barriers, and explains how Together4RD is seeking to move the needle here, by learning from case studies, exploring frameworks for collaboration, and launching pilots to explore how best to plan and deliver multistakeholder interactions addressing real research needs.

**Supplementary Information:**

The online version contains supplementary material available at 10.1186/s13023-023-02853-9.

## Part 1: The EU Context

### Unmet needs of rare diseases and the status quo of European research

Rare diseases (RD) are, by definition, rare; however, there are an estimated 6–8,000 separate conditions classed as rare, based upon the definition espoused by Regulation (EC) No 141/2000, with an average of 4–5 new conditions described every week. This means that collectively, rare diseases affect a significant proportion of the population, approximately 1 in 18 people. Patients and families typically face challenges in every stage of their journey, from seeking an accurate diagnosis to finding a specialist, participating in research studies and accessing the best available treatment and care. Beyond the clinic, rare diseases tend to impact negatively on all aspects of daily life [1]. One particularly sobering statistic illustrating the extent of these inequalities is that 95% of the conditions classed as rare have no dedicated treatment (and where treatments *do* exist, they tend to address the symptoms and have little impact on the natural history of the disease). These tend to be *very* rare conditions, affecting fewer than 1 in 100,000 people, often poorly understood; indeed, It is acknowledged that 84.5% of rare diseases have a prevalence of less than 1/100,000, yet more than 98% of people living with a rare disease have one of the 390 most common conditions (with prevalence between 1–9/100,000 and 1–5/10,000) [2]. The diseases which do have therapies tend to be clustered around one of a limited number of therapeutic areas (60% of orphan medicine products designations during the period 2010–2020 were for oncology, alimentary tract & metabolism, and musculoskeletal & nervous system disorders [3]). All of this results in significant inequalities for patients and their families, because of the rarity of the disease, whilst also posing challenges for healthcare professionals and health systems at large (which often struggle to provide expertise across the heterogeneous range of rare conditions).

The extent of these challenges have marked rare diseases out as an area of priority action at European level, for many years. Key policy documents were issued in 2008 (the Commission Communication on *Rare Diseases: Europe's challenges* [COM(2008) 679 final] [4]) and 2009 (the Council Recommendation on an action in the field of rare diseases (2009/C 151/02) [5]). These landmark policies built upon the regulatory incentives engendered by the 2000 Orphan Drug legislation (Regulation (EC) No 141/2000) to call for national action alongside key European efforts to advance diagnostics, treatment, care, research and social support for rare diseases. Much was achieved in the following decade [3, 6]; however, notwithstanding these achievements at both European and national level, the day-to-day reality for too many people living with a rare disease has sadly changed little. Major unmet needs remain, which can only be addressed through a seismic shift in the way in which research, care and social support are organised, in Europe and beyond. In recent years, much attention has been focused on where the RD field should go next—how can we stimulate new R&D for the thousands of conditions without *any* treatment options (and indeed any basic research activity), whilst also ensuring that therapies developed for conditions benefiting from a relatively strong research interest deliver meaningful and transformational change?

An important attempt was made to revitalise European rare disease policy in 2018, when the European Parliament called for a pilot Project to conduct the first Foresight Study dedicated to rare diseases. The 2-year Rare 2030 project, which eventually ran from January 2019 to Spring of 2021, was led by EURORDIS (Rare Diseases Europe). Rare 2030 aimed to stimulate the development of a new European policy framework to ensure meaningful change for the future ahead, and generated an ambitious set of recommendations [7] to guide Europe towards the future scenarios deemed most desirable. Rare Disease research, in particular, needs to operate within a supportive Research and Innovation ecosystem—it is therefore important to note that in recent years the Orphan Drug Regulation (EC 141/2000) has come under scrutiny. In 2017, a 10-year evaluation report on the EU Paediatric Regulation was published [8] which concluded that the Regulation had provided positive results overall in terms of paediatric product development, but that development for rare paediatric diseases, which is in many cases equally supported through the Orphan Regulation, often failed to materialise.

The last few years have therefore seen significant European attention placed on the challenges remaining for the approximately 30 million people living with a rare disease in Europe. The various projects and sets of recommendations launched to address the gaps and shortcomings of past activities and investments are now culminating in a renewed attempt to garner political support to actually put some of these recommendations into action.^1^ This momentum around rare disease is increasingly noted not merely within Europe, but at the global level.^2^

These sorts of conclusions and policy initiatives are particularly important, given the worrying trend that R&D in Europe is increasingly lagging behind that of other parts of the world, seeing less investment and lower levels of clinical trial activity; for instance, whereas 41% of R&D investments across the board were centred on Europe in 2001, this has now dropped to 31% [9]. It is imperative that Europe regains a competitive edge, especially in terms of research and innovation for *rare* disease, given the major unmet needs. The advantages of working with the pharmaceutical industry, in particular, must be recognised by policymakers working in rare disease –private sector involvement generally remains a prerequisite for successful drug development in this complex field [10, 11]. The in-house knowledge that drug developers hold (particularly around clinical trial execution, regulatory pathways and data), together with their access to financial resources, may be lacking in the (often publicly funded) clinical networks focused on administering care.

Amidst this plethora of recent initiatives, reports, recommendations and policy asks relating to rare diseases, the ERNs are frequently cited as central infrastructures of unique importance: they could evolve to become the foundation of a future European health and research system for rare diseases, by maturing current collaborations into innovative partnerships, thus becoming powerful agents of the change which is so greatly needed.

### ERNs and the potential they offer

#### The concept and origins of ERNs

ERNs are arguably the single most important innovations in health and research for rare diseases in Europe, if not globally [12]. Officially launched in 2017, ERNs were intended to connect European centres of expertise in specialised healthcare fields necessitating a concentration of expertise in order to increase knowledge and build professional capacity across the healthcare and research spheres [13]. Rare disease, though not the sole focus of ERNs, is the most natural and most significant beneficiary. The concept of an ERN was developed by a RD TaskForce Working Group, and gained traction under Article 12 of the 2011 Directive on the Application of Patients’ Rights in Cross-Border Healthcare (the so-called ‘Cross-Border Healthcare Directive’)[14]. 24 ERNs were approved in the first call [15] representing over a decade of preparatory work [16]. These ERNs have been founded as patient-centred networks, where patient participation is fully integrated in the network governance structures and activities, as recommended by the EUCERD recommendations and Addendum of 2015 [17, 18].

At their launch, the 24 ERNs brought together over 900 specialist units in over 300 hospitals across 26 countries (25 EU MS plus Norway) whilst also integrating people living with rare or specialised conditions in a meaningful way; at present, approximately 300 patient representatives collaborate as de facto members of the ERNs. Membership of ERNs subsequently expanded, to include (as of 1st January 2022) 1450 full healthcare providers (HCPs) —which may be entire clinics or hospitals or individual specialist units or centres within a larger institution—as well as 155 so-called affiliated partners (centres which do not fulfil all horizontal and disease-specific criteria established by the European Commission and ERNs themselves, respectively, but will enable every country to access the expertise of an ERN more readily). A central pillar of the ERN concept is that collectively, across all ERNs, every rare disease would have a ‘home’—in this way, ERNs would strive to go beyond the networks created by past EU funding, which were dedicated to individual diseases or small groups of diseases (e.g. E-Pilepsy, EU-CHS, EUROMAC etc. [16]) and instead sought to improve diagnostics, treatment, and care for *all* conditions under the rare disease umbrella.

For these reasons, ERNs were envisaged as powerful tools to erode inequalities for patients, firstly by ensuring an all-disease inclusive scope, leaving no one behind, but also by addressing the geographical lottery faced by so many patients. The latter goal is primarily being achieved via one of the ERNs’ central missions of enabling *expertise* to travel, rather than patients, wherever possible. ERNs have dramatically altered the face of virtual, cross-border care for rare disease, developing a system for shared care, specialist advice and second opinions unrivalled in scope and ambition anywhere. The ERNs utilise a shared and bespoke Clinical Patient Management System (CPMS) [19]: to-date, over 2650 panels have been assembled to review patients referred for this kind of shared virtual care under the ERN ecosystem.

The various activities of ERNs are funded from different pots of European funding [20]. Certain Member States are now starting to provide funding for specific coordination activities.

#### ERNs and research

Notwithstanding the importance of these activities which one could class as primarily care-focused, a major source of the ERNs’ potential stems from their mandate to also add value to *research* into rare diseases and highly specialised medicine. *Directive 2011/24/EU on patients’ rights in cross-border healthcare* [14], through which ERNs were founded, stipulates this requirement, as do the legal acts on which ERNs were established: Art. 7 of the Delegated Decision (2014/287/EU) [21] states that “Among the first set of horizontal and structural criteria and conditions, those related to patients empowerment and patient-centred care; organisation, management and business continuity; research and training capacity appear to be essential in order to ensure that the objectives of the Networks are met”. Annex I of the Delegated Decision further stipulates that one of the horizontal criteria (i.e. criteria which all members of any ERN should fulfil) is as follows:

“(5) To fulfil the requirement set out in point (iv) of Article 12(4)(a) of Directive 2011/24/EU (‘make a contribution to research’), the Networks must: (a) identify and fill research gaps; (b) promote collaborative research within the Network; (c) reinforce research and epidemiological surveillance, through setting up of shared registries”

It is fair to say that for most ERNs, there has been less of a focus over these first five years on ‘research’ per se [20]. This is not to say there has been *no* activity here: many—if not all- ERNs launched surveys internally to assess the extent of research across the broad headings with which most are concerned. This was very important, as networks did not exist at the breadth and depth of the ERN headings prior to 2017. A number of diseases already had robust research communities with well-networked expert communities (e.g. Cystic Fibrosis in the rare pulmonary field; rare anaemias and haemophilia in the haematology field, etc.); however, this was not the case for other conditions. ERNs have also engaged in advancing research via participation in a Horizon 2020 initiative called the European Joint Programme for Rare Disease Research (EJP RD), which engaged partners and linked third parties to represent all ERNs. However, the research potential of ERNs has long been appreciated.

In May of 2018, a Joint Action called RD-ACTION, the European Medicines Agency (EMA) and DG SANTE organised a workshop hosted by the EMA, which produced a report highlighting actions that would need to be taken in order for ERNs to begin to fulfil their research potential [22]. This document also elucidated how and why ERNs hold so much potential as clinical research networks and are perfectly placed to add value to rare disease research: the following summary takes this as a starting point but expands upon these areas of potential to illustrate the opportunity ERNs afford.

#### ERNs are permanent infrastructures

ERNs are not time-bound projects, unlike the so-called pilot networks funded during the 1^st^ and 2^nd^ EU Public Health Programmes (whose structures and resources risked falling into disuse once the funding period ended).

Assuming positive evaluations every 5 years, ERNs may be considered permanent structures, making them important stakeholders for partnerships in research of all kinds.

#### ERNs sit at the interface of the research and clinical spheres

The Legal Acts upon which ERNs are based mandate that the Networks provide added-value across both the clinical and research domains. This is essential in rare diseases, where traditionally that line between care on the one hand and research on the other has, of necessity, been somewhat blurred. All 1450 HCPs participating in ERNs as full members should possess clinical expertise in at least some of the conditions underneath the grouping of that ERN, but should also be research-active, boosting the potential for multicentre trials in Europe (and also for rapid transfer of promising preclinical research into Industry-supported trials). The proximity of research spaces and the clinic is a major strength of the ERN model, facilitating the generation and translation of knowledge and best practice.

#### ERNs are designed to ensure comprehensive disease (and specialised procedure) coverage

When applications for the first 24 ERNs were encouraged to establish their Networks based upon a list of suggested Thematic Grouping [23]: this was to ensure that collectively, all rare disease would have a ‘home’ under at least one ERN. In actuality, many ERNs followed this suggested Groupings schema very closely, and consequently the vast majority of conditions classed as rare were covered by the 24 Networks created under the first call, along with several less disease-focused ERNs more dedicated to specialised *procedures* and areas of medicine in which a concentration of expertise is also of paramount importance (e.g. ERN-TransplantChild). The fact that ERNs are founded upon this principle of inclusion of all rare diseases is a major benefit and holds real potential for research of the future. The reality, as mentioned above, is that within each ERN there is often a ‘focal’ disease or group of diseases which has attracted a relatively large amount of research attention (predating the ERNs’ foundation), and/or is better understood and supported in terms of diagnosis and care (although many ERNs are seeking to address this, for instance by ensuring their registries collect data on all diseases covered by the Network).^3^ But nonetheless, the inherently egalitarian nature of the ERN model is, in itself, a strong step in the right direct of casting much-needed light on the many thousands of so-called neglected diseases which have traditionally lacked any research interest.

#### Data generation/linkage and digital health opportunities

ERNs provide unprecedented opportunities to collect and share high quality, relevant, and interoperable data. People living with rare diseases have affirmed their desire for their data to be reused, to generate new knowledge and understanding, in order to help the next generation of people affected with their condition [25] —so the goal of maximising the use of precious rare disease data is widely supported. The Networks are based upon centres which have demonstrable expertise in particular areas, but the Networking *tools* and platform which connect these well-established centres are being created—or at least delivered—anew. This offers exciting opportunities for the 1450 HCPs (plus 155 affiliated partners) across Europe to subscribe to best practices around creating, collecting and pooling precious rare disease data in a timely manner, to support the provision of highly specialised care and advance research. The CPMS has already resulted in more harmonised and interoperable data being collected for specialist virtual reviews. But the area of registration holds possibly even greater potential in terms of advancing research and understanding [26, 27].

**Fig. 1:**
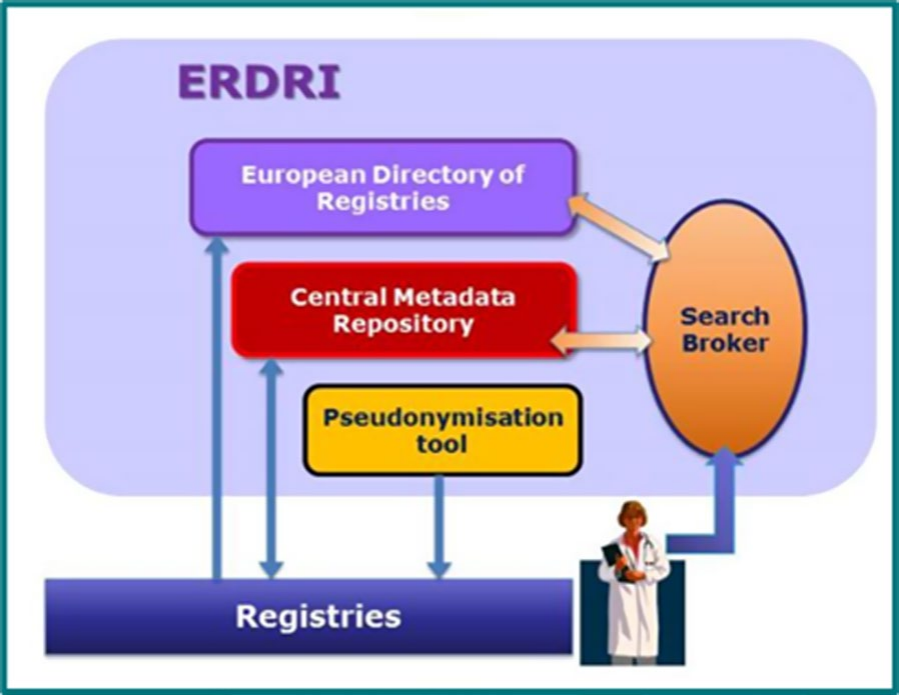
Image used with permission from the Joint Research Centre [31]

DG SANTE had provided funding for all ERNs to establish new registries and/or link existing registries in their fields. Registries are essential tools for generating knowledge about rare diseases, and—depending on the data collected, and its quality—can serve multiple purposes [27, 28]. It is reassuring to see that, notwithstanding the variety in scope of the 24 ERNs, the ERICA (ERN Research Coordination and Support Action) [29] and EJP RD [30] projects have initiated cross-ERN collaboration on registries and health data management tools, processes and policies. At the same time, the European Platform on Rare Diseases Registration, initiated in 2013 by the European Commission's Joint Research Centre (JRC) in collaboration with DG SANTE is building tools to facilitate access and re-use of RD registry data, via its European Rare Disease Registry Infrastructure (ERDRI) (Fig. 1).

The creation of a European platform to increase the reuse potential of precious rare disease data is of major importance when one considers that over 800 rare disease registries exist in Europe (or are fed by European centres or actors) [32]. However, the creation of ERN registries—or platforms to link new ERN registries with historical or possibly new disease-specific registries—holds major potential for advancing knowledge and better care, but also naturally for stimulating and advancing research. Supported by projects like the EJP RD and ERICA, attempts are being made to ensure a certain level of interoperability in terms of the data collected in these new ERN registries. For instance, the Common Data Elements issued by the EU RD Platform have been turned into a richer data dictionary under the EJP RD [33]: this is just one example of efforts to make registry data FAIR (Findable, Accessible, Interoperable, and Reusable). Greater value will come with the advance of individual ERNs agreeing and standardising domain-specific datasets [34].

The summaries above are far from exhaustive, but illustrate why ERNs hold such significant potential to advance rare disease research. However, to-date, the research activity of the ERNs has been limited, for several reasons.

## Part 2: Understanding constraints on ERN Research to-date: and in particular, barriers to Industry collaboration

### What factors have limited ERN-led research?

#### Contrasting priorities in the early years of ERNs

The relatively limited research activities of the ERNs to-date can partially be explained simply by the amount of time required to launch the networks and set up the necessary governance and operational structures, along with the apparent prioritisation (naturally enough given the fact that the ERNs were initiated under the aegis of DG SANTE, not DG RTD) of more overtly care-related activities. However, this does not mean than no ERNs have been active in the research domain. The surveying of the status quo as outlined above, the creation of intra-ERN working groups for research (plus a cross-ERN working group), engagement as key partners in the EJP RD—and more recently the ERICA project –are all important achievements.

#### Lack of suitable funding

One barrier often reported by the ERNs themselves is the notable lack of dedicated funding for collaborative research projects. More recently, funding for the development of the registries, the emergence of the EJP RD mobility exchange programme for ERN researchers, and the launch of ERICA (in 2019) have gone some way to address this shortfall, but there is still no specific funding scheme to foster direct clinical research activities within and across ERNs.

#### Some ERNs represent communities with a limited research track record

Most ERNs would agree that they have not yet scratched the surface of what might be achieved in the research space [20]. And here, the situation is actually quite dramatically different from network to network. Some ERNs have very limited research activity, which reflects the broad communities they represent (i.e. there is limited research in the Thematic Grouping with which that ERN is concerned). For these ERNs, therefore, the persistence of that traditional lack of research activity and momentum over the past 5 years is itself a barrier, as little has come along to incentivise (or indeed enable) more research in the field. Projects such as ERICA are seeking to build capacity across the board, for instance by creating a much-needed repository of PROMs (Patient-Reported Outcome Measures) relevant for rare diseases [35]. However, substantial additional resources and funding are needed to transform these less research-active ERNs into research-ready networks. At the other end of the scale though, it is acknowledged that some ERNs emerged from notably research-active communities, often bolstered by research in a handful of focal disease or disease groups if not by research across the board. Those ERNs, therefore, may report limited research activity *as an ERN* whilst acknowledging that the researchers and centres of which that Network is composed are *highly* engaged in research of all kinds.

#### Confusion in defining research activity ‘of an ERN’

This raises another possible challenge for ‘ERNs and research’, namely, simply defining what constitutes ERN research. This has been a source of some confusion since the creation of the Networks: how to distinguish the achievements of a given ERN, collectively, from the day-to-day achievements of its component centres (and, at a still more granular level, of the individuals involved in that ERN)? Definitions were created in an attempt to alleviate confusion and ensure all ERNs were reporting their activity in a comparable manner [36]. It may be, therefore, that confusion over what *constitutes* research activity of an ERN has actually hampered so-called ERN research.
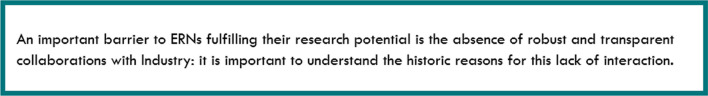


### Barriers to ERNs and Industry Collaboration

“ERNs are still on a learning curve in terms of collaboration and so engagement with all stakeholders to explain the value of collaboration is important” (Franz Schaefer, Coordinator of ERK-NeT, the ERN for Rare Kidney Diseases).

One of the most-frequently cited barriers to ERNs engaging in research is the perceived inability for the Networks to collaborate with Industry—at least when it comes to drug development. This barrier, in itself, is multifaceted. It is true that not all research requires Industry engagement: investigator-initiated research of course takes place, ranging from non-interventional studies to efforts to better understand the natural history of a disease, to trials involving the repurposing of medicines. Nonetheless, given the Herculean task of addressing the unmet needs of the rare disease community, it is very clear that fruitful collaborations between Industry and clinicians/researchers are essential. From the earliest days of the ERNs, however, tangible barriers have limited ERN-to-Industry interactions. Arguably the most significant barrier here is the reticence from the ERN Board of Member States (BoMS), which was set-up via the ERN Legal Acts to oversee the Networks.

#### The scope and significance of the statements from the ERN board of member states

The BoMS has issued two statements (policies, essentially) regarding permissible interactions between ERNs and companies. The 2016 Statement [37] began by acknowledging the fact that Industry plays an important role in improving knowledge of rare diseases and developing clinical tools and therapies. It approves engagement with ERNs ‘where appropriate’ and cites ‘for example in clinical trials and research projects’. Very reasonably, it bars Industry involvement in operational and governance issues (although notably Industry is not singled out but is included here amongst a wider group of ‘external stakeholders’ who are debarred from such roles because there is no legal provision for this). The rest of the statement provides guidance to the Networks on ‘their thinking on engagement with industry’.

This 2016 guidance calls for policies, such as a transparency policy, and for ERN charters defining Conflict of Interest Policies. This Statement was not viewed as entirely prohibitive, in terms of ERN and Industry interactions, but rather stressed the need for careful management of any relationships. Many ERNs were unsure, however, of what –if anything—they *were* permitted to do in this space, and how. Given the fact that all ERNs were faced with tackling the same challenges, a Coordinators’ Group was established soon after the launch of the Networks, and this was complemented by a number of Working Groups on specific topics. These later merged with similar groups which had developed within the BoMS of ERNs. Several Joint policies and documents have been developed through this methodology (e.g. the documents stipulating and defining core indicators for ERN monitoring). One of these Working Groups was dedicated to *Legal and Ethical Issues and relations with Stakeholders*. This body soon began work on a transparency policy and a Code of Conduct. However, the question of precisely how ERNs should handle increasing requests from companies for collaboration of various kinds remained unanswered.

On the 25^th^ June 2019 the BoMS issued an updated Statement [38] concerning ERNs and Industry. The reason for this, it seems, was that lack of “legal provision for the collaboration between ERNs and Industry”, compelled the Board to issue more detailed guidance. This new Statement arguably did little to ameliorate the uncertainty, unfortunately—and where the guidance *was* more explicit than the 2016 document, some points were questioned. With regards to Point 4, for instance, placing emphasis on ERNs seeking public funding before accepting private funding: the scarcity of public funding available for ERNs (and the transient nature of the grants available) has been a constant and significant source of frustration for many ERNs [13, 20]. The suggestion of seeking Industry funding only when more than one company was involved in an activity presented some challenges in terms of the *type* of collaboration that would fit such a set-up (and the relative scarcity of examples of such funding, beyond IMI-type grants etc.).

Point 5, however, was perhaps the most contentious (the bold emphasis is not present in the Statement itself): direct Industry funding was debarred from *“any type of activity relating to the development of diagnostic and clinical practice guidelines (CPGs) or any other clinical decision-supporting tools, development of outcome measures as well as establishing and maintaining patient registries”*. Now, avoiding Industry involvement in the creation of CPGs or similar tools is reasonable, given the potential for conflict of interest.^4^ The stance on registries is interesting, however. As above, all ERNs have received grants of €200,000 over a period of 3 years, to establish a new ERN-wide registry and/or to link existing registries. This is generally considered to be an inadequate sum of money to either create a new and ambitious registry for an ERN, or to address the interoperability considerations by federating existing stand-alone disease-related registries and enabling data collected in different systems with different data dictionaries and access procedures to ‘speak’ to each other.

Following publication of the 2019 Statement, significant uncertainty remained as to how and where (in what activities) ERNs could collaborate with Industry. Most stakeholders (on both the ERN and Industry sides) perceive the lack of clarity on what would be permissible, and under what sort of conditions, as a major barrier to ERNs engaging in substantial research activity. This lack of clarity is accompanied by the restrictions of the BoMS Statements.

#### Conflict of Interest

A key challenge identified for ERN/industry collaboration relates to managing conflict of interest. This is likely at the root of some of the concerns noted to-date in the BoMS, which translated into the cautious wording noted in the two BoMS Statements. The rare disease field is, by definition, small, and it is natural for experts to be approached in their independent capacities by companies to serve on advisory groups or as consultants or similar, and be remunerated for their service. It is of course imperative that people declare their potential conflicts of interest; however, this has always been a reality in this field, and robust examples of Codes of Conduct already exist to ensure ethically responsible professional behaviour of both the experts and companies.^5^ There are also established practices to avoid conflicts of interest in activities for which companies provide financial support to non-ERN networks or stakeholder associations.

#### Privacy and ethical concerns

Unfortunately, collaboration with the private sector is not always viewed in a positive light. If a company is deemed to have acted unethically, particularly if perceived to be at the expense of vulnerable patients, the negative publicity can cause significant damage to the whole R&D field. Rare disease patients are of course particularly vulnerable insofar as sharing their data or participating in clinical research carries greater risks (of identification, for instance). But at the same time, rare disease patients are perhaps more likely to feel pressured into engaging in clinical research than someone with a common disease, as in the absence of a disease-modifying treatment, a trial may be a patient’s only hope: thus the ethical considerations are greater [40, 41]. Patients also express concerns regarding data privacy (despite being overwhelmingly in favour of sharing their data for medical benefits). A 2019 Rare Barometer Voices survey of over 2000 respondents from 66 countries asked rare disease patients if they would feel confident with different stakeholders handling and using their health information carefully: the results for the pharmaceutical industry research were divided, with 45% in favour compared to 50% opposed and 5% unsure (compared to 89% expressing confidence in their physician handling their data) [25]. It is possible therefore that such general concerns on the part of patients have also served to keep Industry engagement with ERNs to the minimum, particularly when coupled with the other factors outlined above. These figures illustrate the need for patients to be in control of their data and consenting.

#### The lack of legal status for ERNs

The ERNs are virtual networks connecting hospital-based units. Each of these institutions are legal entities and the institution that is named ‘Network Coordinator’ is contracted directly by the EC. The Coordinating sites have individual contracts with each of their Members as part of the network governance. However, the ERNs are not legal entities per se. For some of the ERN Coordinators, and occasionally patient advocates, the absence of a legal entity has been reported as a major hindrance to ERNs performing research and collaborating with Industry. This is because a legal entity would foreseeably simplify contracting and other activities necessary for initiating research projects or delivering clinical trials. Similarly from an Industry perspective, if ERNs were legal entities, a company could contract solely with that entity, as opposed to developing agreements with potentially many separate hospitals and universities. However, it must be emphasised that other ERNs do not perceive their lack of legal status as an obstacle, as they are able to use mature policies and procedures at their individual institutions to interact with external stakeholders.

#### Lack of experience in the legal and bureaucratic processes involved in working with Industry

As above, ERNs are at different stages of maturity when it comes to research activity. However, even amongst those with richer, more established research communities, a lack of awareness on how to forge collaborations with Industry may be viewed as a barrier. ERN Coordinating centres are hospitals or universities (or a partnership of the two) —they are legal entities in their own right, but may be ill-suited to tackling the legal, financial and administrative aspects of formally collaborating with a private company. Such institutions are generally very risk-averse, which can hamper attempts at collaboration.

It is possible therefore to identify a range of tangible barriers to ERN-Industry collaboration. However, it becomes apparent that some of these are surrounded by a layer of confusion, and may ultimately be perceived barriers. For instance, solutions exist to manage conflicts of interest (for instance policies and templates have been used by the European Organisation for Research and Treatment of Cancer, EORTC, for many years, in the cancer community), and to address ethical and privacy concerns (robust patient participation in the governance of all relevant activities would be an excellent starting point). Other barriers mentioned here are significant at present, but can be addressed through dedicated and transparent action; for instance, both ERNs and Industry must recognise that even in the absence of the networks themselves possessing legal status, collaborative agreements can be made with key ERN HCPs. However, to utilise such routes to collaboration effectively, support and capacity-building is needed, certainly for the hospitals/units involved, to help them navigate unfamiliar processes, but also for companies engaging in such activity, to seek harmonised procedures which can be replicated in many similar settings. The content of the BoMS Statements, on the other hand, *would* benefit from revisions, to remove uncertainties and ambiguities and generally espouse a more positive and enabling vision of future ERN-Industry collaborations, avoiding clauses constituting unnecessary and impractical barriers.

## Part 3: Methodology and the Added-Value of Together4RD

### How Together4RD is fostering ERN-Industry collaboration

Together4RD emerged from the recognition, on many sides, that a concerted effort was necessary to strategically and concretely address the relative inertia around ERNs and Industry collaborations. It does not seek to advance ERN research per se, in all its forms, as dedicated initiatives already exist for this. Nor does the initiative seek to present itself as the sole forum in which ERNs and Industry can collaborate (Innovative Health Initiative/Innovative Medicines Initiative grants provide the option for public private partnerships, and a new Joint Action on the integration of ERNs into national health systems offers further potential here). Rather, Together4RD aims to ‘move the needle’ and deliver real solutions to some of the key challenges noted above, which to-date have hindered ERNs and Industry engagement and thus setback the pace of progress in rare disease research at large.

**Fig. 2:**
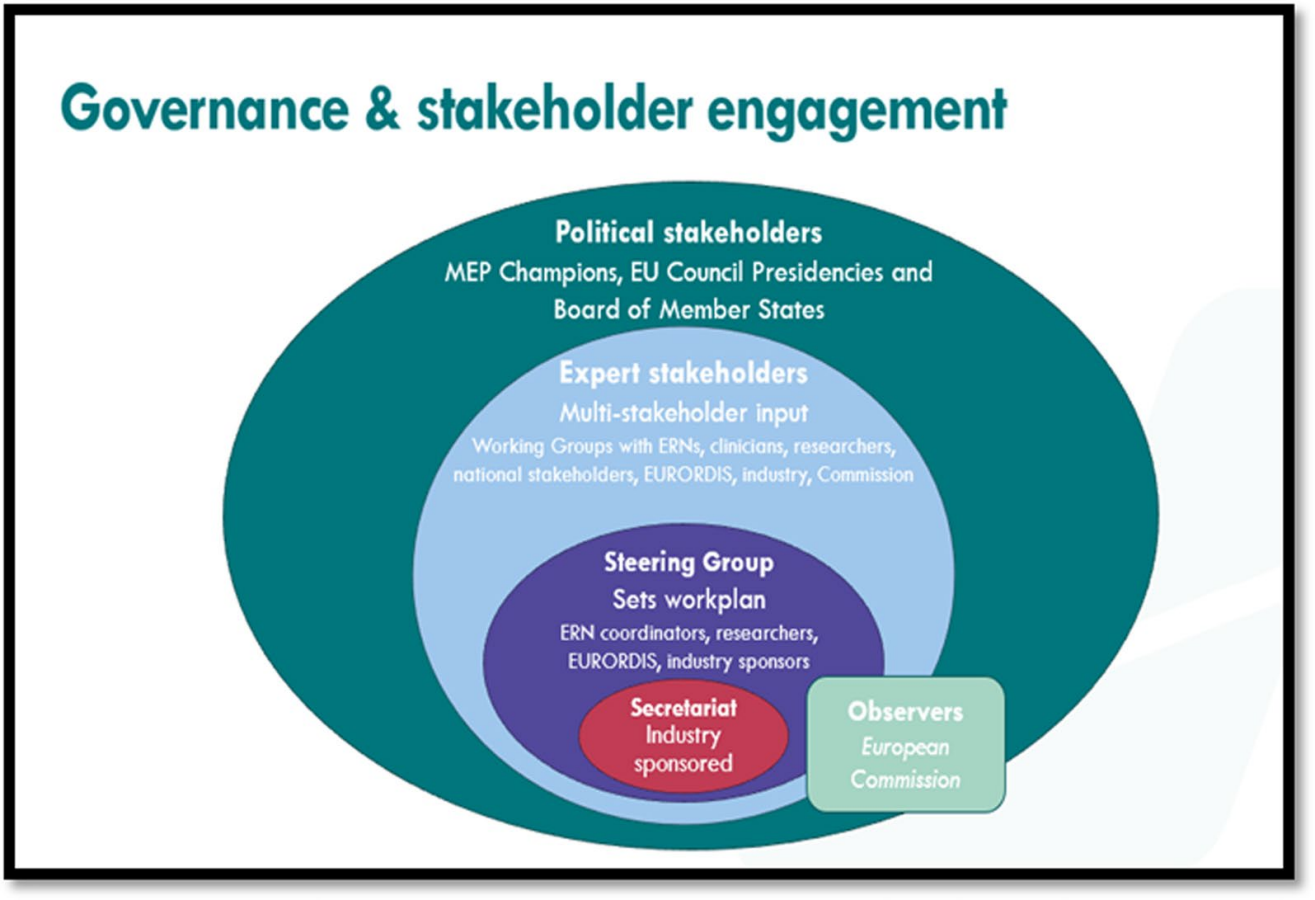
The structure of Together4RD

Launched in December 2021, Together4RD is led by a multi-stakeholder Steering Group, comprising Coordinators and managers from 4 ERNs, pharmaceutical industry representatives, members of the research community, and the European Organisation for Rare Diseases (EURORDIS).^6^ Given its oversight role for ERNs (which includes holding the secretariat of the BoMS), DG SANTE acts as an observer. The Steering Group’s role is to provide strategic input to, and oversight of, all work undertaken in Together4RD, and to promote this through their own networks. The Steering Group is supported in the day-to-day implementation of Together4RD’s work by the Together4RD Secretariat (provided by FIPRA International). The Secretariat is aided financially by funding partners the European Federation of Pharmaceutical Industries and Associations (EFPIA), the European Confederation of Pharmaceutical Entrepreneurs (EUCOPE), Alexion Pharmaceuticals,^7^ Novo Nordisk, Sanofi, UCB and Takeda (Fig. 2).

The core aim of Together4RD is, firstly, to uncover and expose the barriers that exist to more ERN-industry collaboration; and secondly, to offer solutions and structures to overcome those barriers, and unlock potential. Securing political buy-in has been an important pillar of this work, and the initiative currently has four MEP Champions (Frédérique Ries (Belgium, Renew Europe), Sara Cerdas (Portugal, S&D), Ondrej Knotek (Czechia, Renew Europe), and Stelios Kympouropoulos (Greece, EPP)) within the European Parliament that have helped to amplify this vision for a new landscape for rare disease innovation.

### Possible frameworks to guide ERN-industry collaboration

**Fig. 3:**
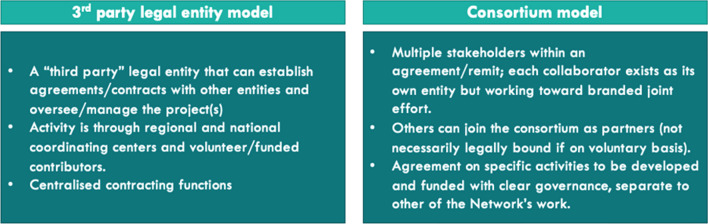
A 3rd party legal entity model vs. a consortium model

Together4RD conducted research in 2022 into possible frameworks to structure ERN collaborations with Industry. As noted elsewhere in this Position Statement, stakeholder views differ with regards to the desirability of ERNs becoming legal entities. It may be that in future, each ERN will become a legal entity (LE) of its own, although at present this is highly unlikely. Or perhaps, a neutral third party will be appointed (this could be a foundation, for instance) to oversee the contracting activities between individual ERNs or groups of ERNs, on the one hand, and single companies or groups of companies on the other. The fact is that neither solution will appear overnight, however; therefore, if pilots are to be launched sooner rather than later, it will be necessary to work within the possible frameworks available to the European rare disease community at present. The key point in both of the above is that ERNs are not legal entities, but the centres (HCP or ‘affiliated’ partners) of which they are composed ARE legal entities (Fig. 3).

One option would be to conduct ERN-Industry engagement via a Consortium Model. Here, a number of independent institutions collaborate based on a particular remit. An example could be the Rare Impact Phase 2 project, in which EURORDIS acted as chair and participated—supported by Dolon which acted as secretariat- alongside 17 gene and cell therapies, Fondazione Telethon, Alliance for Regenerative Medicine and EUCOPE. Individual institutions within the Consortium will be legal entities, but a legal framework is not required for them to collaborate, making this a more fluid and dynamic model. Contracts are still required if any resources are transferred between institutions. Such a model could be possible under the existing frameworks for ERNs, as an ERN HCP such as the coordinating centre or a key research centre could take charge of contracting and making agreements with a company or companies, on the one hand, and also with several or possibly even *all* of its fellow ERN HCPs/affiliated centres, through agreements. This model would of course carry financial and resource implications for the coordinating ERN, which would need to be accounted for.

An alternative would be to embrace a true Third Party Legal Entity Model. Here, one LE—outside of the ERN—would establish agreements and makes contracts with other entities and would oversee/manage the project(s). This could be a new body, in the form of a common research office, for instance inspired by the US RDCRN (Rare Diseases Clinical Research Network [43]) model or the EORTC. Whatever its form, it should be able to support all ERNs in their research activities, which hosts and provides expertise and research capacities as well as overseeing agreements and contracting and managing the financial and legal elements. Alternatively, it could be more like a foundation, serving the needs of all ERNs. A variant on this theme would be to envisage separate foundations or other forms of 3^rd^ parties, playing such a role for each ERN individually. When considering a 3^rd^ party Legal Entity Model, however, it is important to note that some public institutions do not allow this kind of contracting.

Perhaps this should not be viewed as an either/or situation—different sorts of framework may work better for different sorts of activities. It may be that some activities which can be envisaged between ERNs and companies would only ever require the movement of funds to a single centre within that ERN—e.g., if one ERN HCP was tasked with elaborating or expanding a platform to integrate standalone disease-specific registries, or to develop a cross-ERN platform for post-marketing surveillance; in which case, a single contract between a company or group of companies could be made with that single HCP which would deliver the work with the guidance and scientific or clinical support of the wider ERN. However, if a piece of work entailed working with multiple HCPs, each of which required specific resources, there would be two options:

1. A single HCP, which is a LE (e.g. the coordinating centre) contracts with a company/several companies, and that centre then distributes funding to other HCPs/affiliated centres, as appropriate. The onus is then on the ERN Centre to arrange for payments and coordinate activities within the arrangement (which may or may not require an amendment to the contracts Coordinators already have with their HCP members). The bureaucracy and resource implications involved here may or may not be acceptable: such activities can be time-consuming and are not always successful. It is notable, however, that some ERNs have recently started to operate in this way, insofar as a coordinating centre provides funding from grants to individual member centres (e.g. to enter data in the new ERN-wide registry).
2. A company/companies would develop contracts with each of the multiple HCPs it wishes to work with directly, as part of an overall pilot or piece of work. Here, the onus is on the company, and on each of the individual HCPs participating to research. This model has the disadvantage of creating different contracting arrangements across multiple individual HCPs and institutions.

### Learning from case studies: extrapolating lessons and good practices for ERNs and industry

From the 2016 and 2019 Statements discussed in 2.2, it appears that at least some members of the ERN BoMS harbour concerns over the prospect of collaborations between the ERNs and Industry. Although such concerns may not reflect the majority of the BoMS members, the traditional *modus operandi* of the BoMS has been to seek full consensus on the wording of key documents and policies pertaining to the ERNs. This means that the concerns of a few countries can, in theory, have a major impact on the research prospects of ERNs. This is not to suggest that public–private interactions should not be subject to the highest possible ethical and legal standards: the consequences for the whole R&D community, if there is any action that is seen to transgress or act unethically, can be severe and long lasting. What has perhaps been overlooked in past discussions concerning ERNs and Industry, is the extent to which interactions between rare disease clinicians and researchers, on the one hand, and companies on the other, take place every day—and have been taking place, in some cases, for decades, without issue, whilst providing myriad benefits all round. As noted in 2.2, in such small and specialist communities, it would be impossible for leading experts and Industry representatives *not* to be acquainted somehow.

A key milestone in the Together4RD mission to understand perceptual barriers to collaboration and identify workable solutions involved issuing a call for case studies. Examples were sought -via the Steering Group and their wider networks- of instances where Industry has collaborated with a network (largely predating ERNs or existing outside of ERNs), or other body of clinicians, to achieve a particular goal. These case studies summarised what was achieved/is being achieved in each example, what steps were taken to forge this relationship with the company/companies, the lessons learned, and the results of the collaboration. These case studies generally focus either on registries or on broader activity to support clinical research (excepting clinical trials themselves). For the full list of summaries, see Additional file 1: ‘*Case Study Summaries*’.

Two Working Groups were created (involving different stakeholders engaged in the consultative bodies for Together4RD, spanning patient advocates, ERN representatives, Industry, and researchers), to analyse each set of case studies via discussions with the experts involved in setting-up and maintaining them, in order to extract the most pertinent learning lessons and good practices. These Working Groups were met twice, remotely, for 1.5 h each time, and worked via Sharepoint documents.

A range of distinct *types* of collaboration emerged. Together4RD was able to distil these insights and practices into tables, one for each Working Group, to show clearly and comprehensively.

- Firstly the different sorts of collaborations possible with Industry;
  - on the ‘Registries’ side, these include accessing registry data to elucidate natural history, to conduct post-marketing surveillance, to serve regulatory purposes as Real-World Evidence (RWE), and collaborating on the definition of datasets.
  - In terms of broader ‘Clinical Research’, activities ranged from strategic fora to advance research to creating opportunities for researchers to pitch ideas to companies: and from creating or improving biobanks to diagnosing patients through electronic health records.
- And Secondly, to match examples of each activity to achievements of the different case studies, to show how some of these Industry collaborations have been approached to-date.

In generating these tables, and affirming the contents, the experts were encouraged to broaden their thinking from purely what has gone before, to what *ERNs*, specifically, could do in partnership with Industry. These tables are included as Additional file 2: ‘Table showing a range of exemplar collaborations concerning registries, which could be envisaged between ERNs and Industry’ and Additional file 3: ‘Table showing a range of exemplar collaborations concerning clinical research, which could be envisaged between ERNs and Industry’.

It is illuminating to consider how some of these case study entities have managed to deliver these kinds of activities, and to explore the kind of legal frameworks they have utilised. A long-running example of a network (founded in the pre-ERN era) which has collaborated with Industry in multiple ways is TREAT-NMD. TREAT-NMD was established back in 2007, via an FP6 grant, as a network to advance trial-readiness in all neuromuscular diseases. It has created a suite of tools and activities to achieve this goal, and in 2019 was ‘spun out’ of the University which coordinated it, as a legal entity. Key resources include cell and animal standard operating protocols (preclinical research); an advice service (TACT), global patient registries, ethical framework and care guidelines, and family guides, to help develop and extend translation research in the field. Many of these activities have involved Industry, and ethically-robust practices and codes have been developed to facilitate this. One key area of Industry engagement concerns patient registries. TREAT-NMD links numerous registries (and developed core and expanded datasets to standardise data in these standalone registries) to facilitate the identification of specific patient groups and boost patient recruitment. It also coordinates global patient registries for several NMDs. The inter-connected registries provide a wealth of information and can be queried by academic sites (free) or by Companies (for a fee). In the days before it became a legal entity, contracting was performed by the institution coordinating the network, namely Newcastle University in the UK. This was not always straightforward, but procedures were honed over the years to govern the collaborations with Industry, based on policies agreed by the whole consortium (composed of mainly academic institutions and patient organisations). A typical activity entailed registry data being sought by a company, for instance to assess the feasibility of conducting a clinical trial in a given neuromuscular disease. The registries associated with TREAT-NMD were largely national, standalone autonomous registries for conditions such as Duchenne Muscular Dystrophy, which had agreed to collect a common data set defined by the TREAT-NMD consortium. TREAT-NMD established an advisory board composed of the curators of these autonomous registries, called the TREAT-NMD Global Database Oversight Committee or TGDOC. A request from a company would be reviewed by this TGDOC and if favourably reviewed, the team at Newcastle University would negotiate a contract with the company. Aggregate data would be collected by the national curators of each registry, by disseminating a questionnaire via their registries (or simply providing the aggregate data themselves) —a typical query might be ‘how many patients with an X deletion of Y neuromuscular disease are enrolled in your registry, between the ages of 5 and 10, and what proportion are still ambulant?’. The aggregate data from each participating registry would be compiled and returned to the company. The fee paid would sustain the posts responsible for managing these collaborations, and would also fund in-person meetings of all the national registry curators (i.e. it would be fed back into the TREAT-NMD ecosystem). In this case, the Coordinating centre of the network took care of all the bureaucracy and the financial and legal contracting. Contracts could be standardised, to make the process smoother when subsequent companies came along with similar requests. This was essentially a consortium model, therefore, and works well if the key institution taking charge of contracting within the network is well-versed in this kind of activity, and is responsible for *using* the funding to sustain the network activities (i.e. in this case, it was not necessary for the coordinating centre to distribute funds to other institutions, which would add an extra layer of complexity).

Another relevant example here is the ERK-REG case study. ERK-REG is the registry of the ERKNet ERN, for rare renal diseases. It was initiated in 2019 and acts as a single core registry for all rare renal diseases. The Registry collects data from the HCPs which are part of the ERN—this is a mix of epidemiological data concerning diagnostics, phenotypic and natural history data, and data to enable continuous monitoring of the diagnostic and therapeutic performance of HCPs (whilst also assessing guideline adherence). It can also be used for the rapid identification of patient cohorts for clinical trials. To-date, collaboration with Industry has included ERK-REG brokering contracts with sites that have patients eligible for clinical trials, and the provision of aggregate data on over 200 paediatric patients receiving a medicine off-label (which was used as supportive evidence for a Paediatric Investigation Plan). Over the first 3 years, 12,661 patients were enrolled, from 41 paediatric and 17 specialised adult units across 20 countries [44]. Here, the coordinating institution, University of Heidelberg, takes charge of the negotiating and contracting function for the ERN, and signs the contract with a particular company. Indeed, they have recently started to distribute funding from European grants to other member HCPs, to encourage data entry in the registry, and therefore the institution is becoming more adept at this kind of activity. A Data Access Committee assesses requests for access to data from Industry, or indeed any other stakeholder. They are then able to make bilateral collaboration agreements with other member HCPs, as necessary (for instance where work is required to gather or analyse data a given site has inputted to the registry platform). This is essentially a consortium model, therefore. However, ERK-REG is exploring the engagement of a 3^rd^ party legal entity, a charitable foundation, which would be able to make contracts with Industry sponsors and ERN HCPs, instead of all activity going through the University of Heidelberg.

Further analysis of the frameworks by which these kinds of case studies enable collaboration with Industry will be important, as ERNs begin to engage with companies under the aegis of Together4RD pilots. The consortium model approach, in which usually a single centre takes charge of contracting, may likely be a logical first step for many ERNs, as there Is no need to select and engage a suitable 3^rd^ party organisation. But thinking longer term, especially where coordinating institutions -or those otherwise willing to broker collaborations involving the wider ERN—are not accustomed to such activities and lack the knowledge and/or resources to play such a role, the engagement of 3^rd^ party models will require careful consideration (and examples such as the POC Club, and the EORTC used in the wider cancer field should be revisited).

### Towards Together4RD pilots

All Together4RD activities across 2022 built towards the selection of a limited number of achievable pilots, intended to partner ERNs and companies to deliver a concrete and specific activity. The process for collecting pilot proposals involved the following steps:

- dedicated discussions exploring the types of activities which have been conducted to-date between networks (typically predating ERNs) and other groups of stakeholders, on the one hand, and Industry on the other (see above, ‘Learning from Case Studies’)
- extrapolating what such activities would look like under an ERN setting, specifically—what good practices could be embedded (and what resources could be leveraged, thinking of data access agreements etc.) and what would need to be avoided, to deliver the activity ethically and effectively
- Seeking input from the Together4RD Industry sponsors and from all ERNs (via communications issued by the Chair of the ERN Coordinators’ Group) on potential pilots that could be implemented in 2022/23

The proposed pilots should all be able to demonstrate a basic level of feasibility and have the potential to illustrate the types of collaboration possible, as well as expose solutions that can be adopted to address the concerns raised in concerns raised in the section ‘Barriers to ERNs and Industry Collaboration’—particularly in relation to conflict of interest, governance and transparency. Certain considerations were deemed especially relevant for the feasibility and added-value of potential pilots (Table 1):

**Table 1:** Considerations for potential pilots

Clarity of goals and expected outcomes	Potential for scaling up to other ERNs
If there is already an established infrastructure in place	Proposal has patient group support, specifically from the patient group involved in the ERN or ERNs that would participate in the pilot
Resources available/ committed	Involvement of smaller ERNs
Single to multiple company pilot	Has potential to satisfy BoMS criteria

A broad range of possible pilot-type activities has been elaborated by Together4RD, as per the previous section, ‘Learning from Case Studies’, and can be found in Additional files 2 and 3.

A range of concrete pilots will be selected, to be spotlighted through Together4RD, in order to expose different collaboration models and approaches. Lessons learnt from the pilots, such as how to manage conflicts of interest, organise governance and ensure transparency, will be extracted and disseminated broadly to ERNs, the BoMS, patients, and the broader Industry community, to present the broad rare disease field with tangible examples of how ERNs, specifically, can collaborate with Industry.

The emphasis is very much on paving the way for further ERN-Industry activity, and in this respect, it will be important to create useful tools and resources, wherever possible. As an estimated 70% of contractual data elements are similar across all activities, the Together4RD pilots will explore the possibility of creating a standardised contract template, agreed by ERNs and an Industry Association. Ideally, such a template will include standard sub clauses for contracting with individual Companies as well as other options for contracting with consortia of multiple Companies.

Pilots which are selected will need to be funded (in terms of all directly incurred costs) by the company or companies which would partner with the ERN/ERNs in question. Together4RD would continue to play a role of neutral broker, monitoring the progress of the projects from a feasibility and operational perspective, with the goal of extracting key learnings and best practices which can be shared and replicated (or indeed, approaches which prove time-consuming or unnecessarily bureaucratic and should be avoided in future activities). However, alongside these more traditional pilots, Together4RD will work towards a pilot involving all 24 ERNs and myriad companies.

“It is important that pilot activities exploring ERN and Industry collaborations are able to provide added-value for ALL ERNs in some way, and do not solely focus on fields which are already reasonably mature, research-wise”

Till Voigtländer, Board of Member States of ERNs (Representative for Austria)

### A pilot for all ERNs

It is acknowledged that due to the historical barriers limiting ERN and Industry discussions, Industry representatives are generally unable to participate in ERN meetings or workshops: representatives of companies occupying strategic positions, e.g. in EFPIA or EUCOPE, are involved in—and indeed help to shape the development of—projects or initiatives in the pre-competitive space, and are thus very familiar with ERNs and their potential, but there is limited engagement between individual ERNs and companies, *as* ERNs. It is also likely that many companies—especially smaller biotech companies lacking a European foothold, perhaps—are generally not aware of what ERNs actually offer nor what they have achieved and what priorities they are embracing for the coming years. This is a major gap to collaboration. It is also recognised, however, that some ERNs represent fields which are not particularly active in research generally, and thus their HCPs and individual experts perhaps do not have vast networks of professional connections and do not receive requests for collaboration. As in all ERN activities, it is critical to avoid favouring certain ERNs over others, as this would mean favouring certain diseases or disease areas over others. For these reasons, Together4RD stakeholders are interested in developing a pilot focused on a forum (or fora) or some sort, which will serve to address the gaps but in a way that will benefit all ERNs. For instance, Together4RD could envisage creating a dedicated space (i.e. beyond simply inviting Industry to a conference) for representatives of ALL ERNs and companies to come together. Perhaps a dedicated session could be included at the beginning or end of official EC Conferences. Alternatively, Together4RD could establish an ERN-Industry strategy forum, something similar perhaps to the EURORDIS RoundTable of Companies [45], in which ERN Coordinators/their research leads meet once or twice a year with Industry representatives and patient representatives to strategically discuss a subject of mutual interest, from a general (i.e. cross-disease) perspective, of interest to many or all Networks. A tier above this, Together4RD could create a new/utilise an existing forum to support more *specific* and *involved* discussions between Industry and INDIVIDUAL ERNs. This would not necessarily need to be mutually exclusive with the previous forum idea—one could envisage a shared event, which then focuses down and splinters into ERN-specific sessions, each involving representatives of the companies most interested in/active in the area with which that ERN is concerned. Alternatively, ERN-specific fora could be organised as entirely separate and distinct meetings, following the mould of the ACCELERATE initiative in the paediatric cancer field (see Additional file 1).

## Part 4: Conclusions, recommendations, and policy asks

Over the course of 2022 and 2023, Together4RD has refocused attention on the lack of collaboration between ERNs and Industry (which, as explained, impacts negatively on the research potential of the Networks): assembling steering groups and multi stakeholder meetings; seeking and analysing case studies of activities to serve as precedents for ERN and Industry collaboration; initiating discussions on good practices and practical solutions to barrier, real and perceived; and most recently, initiating a call for pilots—all of this constitutes invaluable groundwork to revisit this important topic and drive the rare disease community a step closer to fulfilling a fundamental recommendation issued by the Rare 2030 Foresight Study:

“Clear rules are required that enable European Reference Networks to collaborate with industry across a range of pre-agreed activities, clarified and tested through pilots, using shared SOPs to accelerate research and build mutually-agreeable public private partnerships: a central business development/tech transfer office could promote, coordinate and supervise European Reference Networks interactions and agreements with industrial partners” [7]

**Fig. 4:**
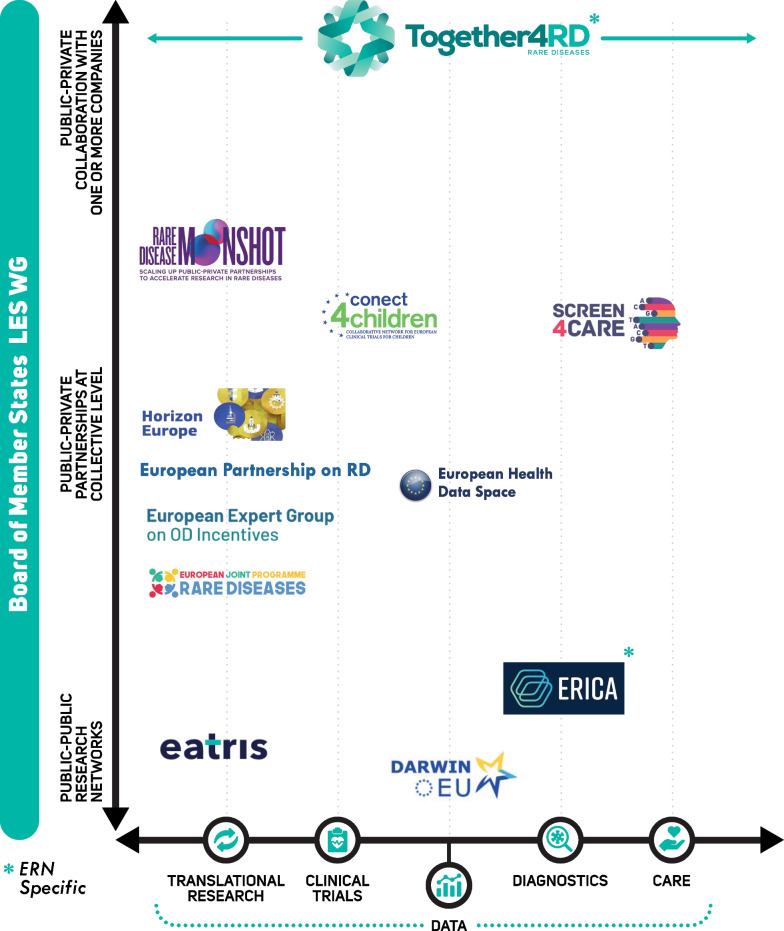
Initiatives of relevance to Together4RD and their positioning within the European research ecosystem

Together4RD has essentially assumed some of the key functions called for in this recommendation and has joined other initiatives in working to address multiple barriers to ERN-Industry collaboration—Additional file 4: ‘Overview of other initiatives complementing the work of Together4RD’ summarises the most relevant of these, and Fig. 4 illustrates how these initiatives interlink with research of various types, at different stages.

As explained, particularly in section, ‘Barriers to ERNs and Industry Collaboration’ above, the barriers to fruitful ERN-Industry collaboration are both numerous and multifaceted. Table 2 summarises how Together4RD is planning to address each of these barriers.

**Table 2:** How Together4RD is addressing barriers to ERN-industry collaboration

Barrier to ERN-industry collaboration	Solutions in development by Together4RD (or others, as appropriate)
Concerns on the part of some members of the ERN BoMS concerning Industry and ERN interactions: stemming from a lack of awareness of the nature such collaborations can take, and of the current extent of successful collaborations outside of the ERNs	Development of rare disease case studies to illustrate a range of examples in which networks (at national and international level) engage/have engaged with Companies for particular purposes
	Disseminating these case studies to the BoMS, to show that win:win collaborations indeed exist
Concerns from the BoMS (and indeed from ERNs and patients themselves) over conflicts of interest	Initiating ERN-Industry engagement via Together4RD pilots, using the Code of Conflict developed by the WG on Legal and Ethical Issues, should appease concerns
Possible lack of awareness or clarity on the range of activities which could take place between ERNs and Industry	Utilising the case studies to more clearly distil the sorts of activities which could feasibly occur between ERNs (specifically, moving forwards) and Industry, to support the development of palatable pilot proposals the BoMS members would support
Concerns amongst BoMS that opening the door to ERN and Industry interactions would only benefit a few of the already more research mature ERNs	Together4RD will explore launching a multistakeholder forum to enable ERN and Industry interactions and advance strategic (and increasingly operational) collaborations across the board. Together4RD will, with the stakeholders involved, agree a preferred model for this
Administrative and bureaucratic efforts and time required to contract with Companies (exacerbated in the absence of a readily-available legal entity)	Planned development of standardised templates all ERNs could use (by making use of a designated HCP or Third Party) to contact with single Companies or multiple Companies
The fact that ERNs are not legal entities, and it appears that the European Commission is not seeking to make them so (in the near future at least)	The pilots showcased by Together4RD will demonstrate how either a consortium model or third party agreement may serve to deliver the results ERNs and Companies wish to see. It may be concluded that different Frameworks suit different sorts of activity, but either way, guidance -for now and for the future- will be proposed by Together4RD in the light of the pilot experiences
Inadequate funding for ERNs limits scope and ambition to engage in research in a meaningful way	Under the EU4Health programme, greater financial resources (provided through more amenable and appropriate and less bureaucratic grant processes) is already relieving pressure on the ERN coordination teams, which should serve to stabilise the core Network structures and services. Additional public funding is expected from the future RD Partnership and eventually from Member States to support ERN research activities and data collection. Coupled with a means of obtaining private funding for mutually-beneficial research activities, accelerated by Together4RD, and bolstered by a robust Code of Conduct issued by the Working Group on Ethics and Legal Issues, the hope is that resourcing becomes less of a barrier to ERNs fulfilling their potential
Lack of certainty on the part of ERNs of what they are able to do with Companies, and how to approach different activities	The disease-related pilots selected to be showcased by Together4RD will be closely followed and analysed, to distil good practices and lessons learned, which should serve to optimise all future interactions and should be illuminating for less-experienced Networks
ERNs and their potential are not always well understood—some companies, especially SMEs with limited European traction, are not aware of their existence or if they are, do not realise the breadth and depth of expertise ERNs offer	This Position Statement in itself should begin to raise awareness amongst the broader private sector. Foreseeably the Moonshot (and future RD Partnership, if Industry is able to eventually play a meaningful role) will also serve to boost this awareness-raising. The Together4RD pilot on a forum/fora by which ERNs and Industry can connect transparently and discuss needs and strategies with patients and other key stakeholders, should also address this challenge. And as Together4RD pilots are delivered, presumably increasingly word will spread of the ERNs in SME circles even outside of Europe
Limited basic research and Industry interest in the conditions addressed by a given ERN, which traditionally has therefore had limited research activity	Together4RD will offer benefits here firstly by simply networking the various stakeholder groups involved, and opening up the conversation. A pilot providing either a cross-ERN forum for strategic Industry discussion, or ERN-specific multistakeholder fora, will create a space for all ERNs and thus all disease domains to identify research gaps and meaningful patient-centred needs, and foreseeably make it easier to devise projects to begin to address these and build momentum for neglected conditions (which potentially could be supported through the future RD Partnership, Moonshot or other avenue)

### Conclusions and recommendations on moving from past case studies to ERN-industry projects

As illustrated in the methodology section ‘Learning from Case Studies’, and in Additional files 1–3, the core work on collecting and analysing concrete case studies to explore the strengths and challenges of past and present engagements between groups of clinicians/researchers and Industry, has garnered many conclusions: on the range of activities that could be undertaken for the benefit of people living with a rare disease, and on good practices and approaches which simplified such undertakings. Inclusion of case studies in the annexes to this publication is not to suggest that these are the only examples, nor does it mean that the design and delivery of the activity in each case is perfect or is the only way of working. Rather, they are highlighted in recognition of their demonstrable achievements in developing solutions to work successfully with Industry to achieve concrete goals—and such precedents are invaluable. Not all of these lessons can be replicated in this Position Statement, but they will be used to optimise the delivery of the Together4RD pilots, together with experiences from established consortia used to working with Industry, especially the EORTC in the cancer field and the RDCRN in the US. In this way, Together4RD will seek to match solutions with challenges.

As noted throughout this publication, there is no ‘one-size-fits-all’ approach when it comes to ERNs—needs and realities differ. In analysing the case studies under 3.3 and the Additional files 1–3, it should be noted that some of these resources and models come from fields which are now represented in research-mature ERNs. Where things are working, therefore, in communities like the paediatric cancer, renal, neuromuscular and others, it may be that the goal in some respects will continue to be business as usual (although the prospect of *improving* what such groups do, and doing what they do *better*, via Industry collaboration, is very appealing). In other fields, partnering ERNs and Industry will truly represent perhaps the best means of actually kick-starting research and development, and resources must be built afresh. To ensure Together4RD pilots can demonstrate tangible benefits in particular diseases whilst also serving to move the needle for ALL ERNs, the concept of a pilot dedicated to a strategic forum (or fora), as outlined in the section ‘Towards Together4RD Pilots’ above, will be particularly important.

#### Unlocking the potential of data

It is notable that in considering the most desirable sorts of pilots for ERNs and Industry, important cross-cutting points appear time and again. Many of these caveats concern *data*, the collection, management, standardisation, federation, and sharing of which—whilst always protecting privacy—is of course absolutely critical to unlocking advances in knowledge generation and research for rare diseases. For instance, it is essential that any data-related pilot leverages the advances over the past few years around making data more FAIR. Resources such as data dictionaries and data standards should be reused, wherever possible, and in developing or optimising such resources to better serve pilots, the community must ensure a global outlook (there is little advantage in developing or embracing standards to increase the interoperability of registry data in Europe if the US, for instance, is adopting contrasting and incompatible standards or other assets to optimise syntactic and semantic interoperability). This global perspective is especially important in the clinical research space, as companies tend to operate at the global level—but equally, for the most rare diseases, the critical mass required in terms of patient data can *only* come from a global collaboration.

As a major potential of ERNs lies in the fact that they are nested within leading hospitals, however, ERN-Industry projects will increasingly need to address not only the well-known issues concerning *registry* completeness, interoperability, data quality and suitability for research—increasingly regulatory—activities, but will need to truly push the boundaries of what has been doable to-date and invest in bridging the gap between health and research data. Unlocking the potential of Electronic Health Records (her) data and federating/pooling with other types of data (such as registry data, patient-reported data, clinical trial data, etc.), to support better and earlier diagnostics, elucidate natural history, monitor longitudinal outcomes, and support research and regulatory goals, will require significant resources—human, financial and technological. Even thinking purely of registry data entry, Together4RD’s research has illustrated the extent of data entry challenges (although here at least, possible strategies have been identified. For instance the ERK-REG registry appears particularly successful at getting sites within the rare renal ERN to provide data on their patients, which can then show the clinical activity and outcomes for patients in different HCPs across the ERN. To receive funding, the HCPs need to provide data. The European Society for Blood and Marrow Transplantation registry case study employs both ‘carrot and stick’ approaches.) But unless and until data entry and federation procedures evolve to become significantly more automated, the burden of getting data into various systems will remain a hindrance to advancing diagnostics, care and research. For these reasons, it will be essential for Together4RD pilots to synergise with efforts to implement a European Health Data Space (EHDS).

“ERNs are the cornerstone of clinical research on rare diseases. EURORDIS and the patient community call to establish ERN-industry collaborations under a public-private partnership framework informed by flagship pilots, to harness the research capacities of all partners, making Europe more competitive globally”

Yann le Cam, CEO of EURORDIS

Robust recommendations exist concerning strategies to making data FAIR and indeed to creating or adapting registries broadly—it is important that the first pilots between ERNs and Industry respect best practices and long-term strategic recommendations,^8^ even if a ‘quick win’ is tempting. For instance, developing ad hoc standalone drug-registries has long been viewed as poor practice [46]. Given patient preferences to control consent over what happens to their rare disease data [25], governance issues are crucial, and data access should be maximised, to allow it to serve as many purposes as possible. It is also important to avoid setting-up entirely new registries when options exist to expand or connect existing structures: where a new registry is unavoidable, particular care should be given to interoperability with other key resources.

Therefore, in designing and delivering pilots, it will be important to agree how and where companies can best add-value to the registry landscape, and this ties in to a need for wider discussions on what different activities connected with registries should be supported by which type of stakeholder. There are a range of costs associated with building, evolving and sustaining powerful registries, from establishing the core infrastructure to providing funding for individuals based at different HCPs to actually enter data. The most appropriate roles for the European Commission, for Member State authorities, and for Industry, need to be ascertained. For instance, although some within the working groups felt that funding should be used to support the maintenance of the core registry infrastructure in future (initiated through modest EC funding), the majority seemed to feel that this should remain publicly funded. For some fields, resources are needed to federate disparate ecosystems of disease-specific registries which were often set-up in different ways (before the ERNs) and are not readily compatible, but which hold precious data which should be leveraged by the ERNs and wider community. Is this an activity Industry could support? Then at the other extreme, for some ERNs, disease-specific registries are extremely scarce, and addressing this gap would be extremely beneficial but will require funding. Many experts also see major potential in the concept of Industry collaborating with ERNs to collect data for regulatory purposes (ideally working *across* companies to co-create, develop and sustain *platforms* for newer activities such as post-marketing surveillance^9^)—collaborations of this kind in the pre-competitive space would represent a real change from past investments and mechanisms will need to be developed to make such an enterprise profitable for companies whilst also serving the greater good. There is a good case for Industry support in developing more meaningful and rich modules for disease-specific data, especially where this is expected to serve a regulatory purpose. In all such discussions, it is important also to acknowledge that Industry should not be viewed merely as a source of funding—ERN and Industry pilots should embrace a broader vision of mutual added-value, beyond the purely financial.

#### Managing stakeholder expectations, building trust and consolidating partnerships

Another key conclusion is that meeting the goals of Together4RD will sometimes require compromise. Stakeholders need to be willing to bend from often quite rigidly-held positions and beliefs to meet in the middle in order to move things forwards. People perceive the real barriers to ERN and Industry engagement rather differently, and also sometimes have different views on what the ideal set-up of the future should be: to some, the most meaningful barrier is the fact that ERNs are not legal entities. The question of whether ERNs should or should not be legal entities is impossible to avoid in this work; however, what seems clear is that DG SANTE has no immediate plans to pursue such a course of action, and the field cannot wait for this possible development to commence ERN and Industry engagement. The project therefore committed to launching pilots which can be delivered using the structures and workarounds and frameworks which exist today, as outlined previously. It is necessary therefore to promote robust, real-world examples of what is possible now, without each ERN launching as a legal entity in its own right, whilst continuing to support efforts to better understand what different stakeholder groups actually wish to see (and why), in an ideal future.^10^

Another important dimension of managing often divergent expectations around how ERNs and Industry should collaborate is actually bridging the gap between Industry and the rest of the rare disease community. Often, the realities and needs of different parties are not well understood; in particular, company constraints -and indeed the constraints of drug development generally—are not always clear to those outside of Industry. There is therefore a need to communicate more transparently, on all sides. Patients’ expertise in their own conditions should of course always be acknowledged and properly integrated in research design [47, 48]; to this end, patient representatives in the fields most relevant to the selected Together4RD pilots should partner in their design and delivery, wherever appropriate.

Finally, launching the first pilots between ERNs and Industry will continue to require tactful and responsible coordination and oversight—particularly, perhaps, when it comes to the more sceptical elements within the BoMS. The extent to which the BoMS has the authority to actually debar Industry and ERN activities is unclear—but even if future BoMS decisions are made via a quorum, as opposed to requiring consensus from all member nations on all words in all policies relating to ERNs, the strategy of Together4RD must be to *persuade* and assuage concerns, and to enable national decision-makers to recognise the responsibilities of individual countries to do their part to address the many unmet needs facing people with rare diseases. For these reasons, it is imperative that pilots are properly set-up and monitored by Together4RD—this does not preclude companies from setting up additional pilots with ERNs of their choosing, down the line, but it is important that these first formal engagements are held up as learning experiences, to build confidence and iron-out specific challenges that may arise in delivering the pilots. Based upon the lessons of such pilots, the expectation is that activity between ERNs and companies would continue to expand and grow in future, buoyed by agreed good practices and employing consensus safeguards to ensure mutually beneficial and ethical collaborations moving forwards.

#### Open and transparent engagement between ERNs and industry

There is an often-unspoken irony surrounding the status quo. Some ERNs emerged from quite research-active communities, meaning their Coordinators and constituent HCPs have an established track-record of regular interactions with Industry, assuming various forms. And despite the unease from some European countries around the notion of ERN and Industry collaborations (as exemplified in the 2016 and 2019 BoMS statements) the reality is that the individuals involved in ERNs did not cease their Industry engagement in 2017. Nor should they have done so, when to block vital research would be so injurious to the 30 million people in Europe dealing every day with the burden of rare disease. Instead, they continue their collaborations as individual experts, or as academic institutions, rather than an ERNs. The question of what constitutes Industry collaboration AS an ERN is a thorny one. The present situation is actually deleterious for the entire rare disease field, for various reasons:

- it works *against* the very spirit of open and transparent interaction sought by the companies, the ERNs and the patients and of course the BoMS itself, and actually creates more grey areas;
- it prevents Industry and ERNs from embarking on truly new activities. The sorts of collaborations summarised in part 3 have, in most cases, been happening for years—but truly *new* activities are foreseen which the ERNs are perfectly placed to usher in with Industry. Whereas it may be possible for individual ERN experts or HCPs to continue their ‘business as usual’ collaborations, it is likely that few would feel comfortable embarking on new and substantial projects with Industry which would very clearly be seen as activities OF the ERN, in the absence of a supportive atmosphere such as Together4RD is seeking to provide;
- it perpetuates the differences—and potentially inequalities- in experience between ERNs, as those with robust research communities continue to engage in their own capacities, if not exactly as the ERN, whilst the ERNs representing fields with limited or no research activity will surely struggle to open up opportunities and overcome the relative inertia in a climate in which Industry engagement is somehow frowned upon.

The goal of Together4RD to move the needle here is therefore particularly important. The project should elevate ERN-Industry activity from something which happens in a small number of ERNs by a roundabout route and in a piecemeal way (without being really reported or celebrated) and bring it into the light, to do things properly and more equitably.

#### Leveraging strategic and political opportunities

This vital foundational work should pave the way for further opportunities for ERN-Industry interaction to advance, foreseeably through the much-anticipated Moonshot and the European Rare Disease Partnership. This is the perfect moment to take these overdue steps. For one thing, the political will is there.^11^ Robust recommendations exist (and will foreseeably be implemented through a post-Rare 2030 European Action Plan, a new Council Recommendation, or similar) which stipulate high-level solutions necessary to move the rare disease field forwards in leaps and bounds, as opposed to the incremental (or indeed non-existent) pace of progress seen in so many communities.

Precedents exist, such as the case studies showcased above but also crucially in the form of the US RD Clinical Research Network (RDCRN) [43], for instance, which constitutes an important model for European imitation or adaptation: the RDCRN has grown from its foundation in 2002 to now consist of 20 disease consortia collectively addressing over 190 different rare diseases and engaging ca.400 clinical sites (including around 50 international sites in approximately 20 countries). Crucially, the RDCRN has been able to leverage private funding as well as public support, and as a result has been able to conduct cutting-edge rare diseases research, including gene editing and gene therapy trials. The learnings from this may be very valuable for the European setting [11]. What could then be achieved by the ERNs—which arguably offer even greater potential^12^—if similarly unbridled in the research sphere? The European-level will to unlock the potential of precious rare disease data is greater than ever, and with momentum growing around the EHDS—with the accompanying availability of technical, legal and organisational solutions and assets- there is finally real cause to hope that ambition will translate into reality.

To leverage political support for this important work, Together4RD has issued four Recommendations, in the form of Policy Asks [49], summarised in Table 3.

**Table 3:** Summarising the Together4RD Policy Asks [49]

Recommendation 1: ERN Governance	Recommendation 2: Public–Private Research Collaboration
Promote transparent governance structures and open dialogue to empower and advance ERN—industry collaboration	Create a Forum (or Fora) for public–private exchange of pre-clinical knowledge for ERNs
Recommendation 3: Independent, Well Resourced and Effective ERN Registries	Recommendation 4: EU Rare Disease Action Plan Collaboration
Ensure ERN registries are adequately financed via public funds and remain independent, whilst clarifying & optimising their potential for collaboration	Create a comprehensive European Action Plan for Rare Diseases that supports public–private partnerships

This Position Statement is being launched in the period of the ERNs’ first 5-year evaluation. This is a perfect moment for Europe to take stock of how far the Networks have come, and to embrace an ambitious and forward-thinking vision to guide the rare disease field in Europe and beyond to where it wants—and needs—to be. Although the barriers the Together4RD project must surmount are not insignificant, the consequences of *not* tackling these ingrained issues would be severe—ERNs will continue to be hampered in reaching their potential and Companies will increasingly perceive that the ERNs are not open for collaboration, with rare disease patients the ultimate casualty.

### Supplementary Information

**Additional file 1**. ‘Case Study Summaries’. (Summaries generated to support creation of this Position Statement).

**Additional file 2**. ‘Activities suitable for ERN and Industry Collaboration (Registries)’ (A table of data generated by a Together4RD Working Group to support creation of this Position Statement).

**Additional file 3**. ‘Activities suitable for ERN and Industry Collaboration (Clinical Research)’ (A table of data generated by a Together4RD Working Group to support creation of this Position Statement).

**Additional file 4**. ‘Overview of other initiatives complementing the work of Together4RD’ (Summary illustrating how the mission of Together4RD fits into a broader ecosystem of projects and initiatives working towards a more collaborative ERN-Industry ecosystem).

## Data Availability

Not applicable. Data sharing is not applicable to this article as no datasets were generated or analysed during the current study.

## Abbreviations

BoMS: Board of Member States of ERNs
CDA: Confidential Disclosure Agreements
CoI: Conflict of Interest
CPGs: Clinical Practice Guidelines
CPMS: Clinical Patient Management System
CTSR: Care and Trial Site Registry
c4c: Conect4children
DMD: Duchenne Muscular Dystrophy
DG RTD: Directorate General Research & Innovation
DG SANTE: Directorate General Health & Food Safety
EBMT: European Society for Blood and Marrow Transplantation
EC: European Commission
ECET: European Collaboration for Epilepsy Trials
EFPIA: European Federation of Pharmaceutical Industries and Associations
EHDS: European Health Data Space
EHR: Electronic Health Record
EJP RD: European Joint Programme for Rare Disease Research
EMA: European Medicines Agency
EORTC: European Organisation for Research & Treatment of Cancer
ERDRI: European Rare Disease Registry Infrastructure
ERICA: ERN Research Coordination and Support Action
ERKNet: The European Rare Kidney Disease Reference Network
ERK-Reg: The European Rare Kidney Disease Registry
ERN(s): European Reference Network(s)
EU: European Union
EUCOPE: European Confederation of Pharmaceutical Entrepreneurs
FAIR: Findable, Accessible, Interoperable and Reusable
HCP: HealthCare Provider
IMI2: Innovative Medicines Initiative 2
ITTC: Innovative Therapies for Children with Cancer
JRC: Joint Research Centre
LE: Legal Entity
LES WG: Working Group on Legal and Ethical Issues and relations with Stakeholders
MEP: Members of European Parliament
NH: Natural History
PCOMs: Patient-Centred Outcome Measures
PMS: Post-Marketing Surveillance
PROMs: Patient Reported Outcomes Measures
POC: Proof of Concept
RDCRN: Rare Diseases Clinical Research Network
R&D: Research and Development
RWD: Real World Data
SMA: Spinal Muscular Atrophy
TACT: TREAT-NMD Advisory Committee for Therapeutics
TGDOC: TREAT-NMD Global Database Oversight Committee
WG: Working Group
WP: Work Package

## Footnotes

1 For example, the #30 million reasons campaign, led by EURORDIS, emerged out of Rare 2030 to target the French, Czech Republic and Swedish trio of EU Presidencies in an effort to place rare diseases -and specifically, the outputs of Rare 2030- firmly on the European agenda (perhaps eventually by adoption of a new Commission Communication, Council Recommendation, or an Action Plan, such as has been deployed in the cancer field).

2 Evidenced for instance by the evolution of IRDiRC (the International Rare Disease Research Consortium), the adoption of a UN Resolution on Rare Disease in late 2021, and the WHO signing a MoU with Rare Diseases International to scope a Global Network for Rare Disease.

3 In its position paper on mature ERNs [24]. EURORDIS. Recommendations to Achieve a Mature ERN System in 2030. 2020. Page 33], EURORDIS has called for all ERNs to issue step-wise expansion roadmaps to ensure that networks move away from an initial focus on merely a subset of conditions.

4 That being said, the approach of the BoMS statement may be a little too black and white here: there are examples of Companies playing no role in the *development* of CPGs or Clinical Decisions Support Tools but post-publication funding their translation into different languages and/or their development into accompanying layperson summaries, support the running of workshops and masterclasses for greater implementation of the practices, etc.

5 For example see [39].EORTC. EORTC Code of Ethical Conduct [cited 2023 22nd March 2023]. Available from: https://www.eortc.org/code-of-ethical-conduct/.

6 For a full list of Steering Group members, visit the Together4RD website [42].Together4RD. What is Together4RD—Steering Group [Available from: https://together4rd.eu/what-is-together4rd/steering-group/.

7 Sponsoring company for 2021–2022.

8 E.g. those espoused by the Rare 2030 Recommendations 7.Kole A, and Hedley, V,. Recommendations from the Rare 2030 Foresight Study: The future of rare diseases starts today. 2021., especially 105–6.

9 E.g. “Post-marketing surveillance for orphan therapies should be organised at the European level, through quality-assured shared data registration platforms/disease registries” page 117 of the Rare 2030 Recommendations.

10 It is notable that the question of whether or not ERNs should become legal entities was amongst the most polarising subjects addressed when generating the Rare 2030 recommendations concerning ERNs.

11 See see Part 1, ‘Unmet Needs of Rare Diseases and the Status Quo of European Research’

12 See See Part 1, section entitled ‘ERNs and the potential they offer’ and under Part 2, ‘What factors have limited ERN-led research ?’. Tumiene B, Graessner H, Mathijssen IM, Pereira AM, Schaefer F, Scarpa M, et al. European Reference Networks: challenges and opportunities. J Community Genet. 2021;12(2):217–29.
