# Supplementary material for: Together4RD position statement on collaboration between European reference networks and industry
Source: Orphanet J Rare Dis. 2023 Sep 5;18:272. doi: 10.1186/s13023-023-02853-9 (PMC10478454; doi:10.1186/s13023-023-02853-9)
Supplement: Supplementary file 1 — Additional file 1. ‘Case Study Summaries’. (Summaries generated to support creation of this Position Statement). [file 13023_2023_2853_MOESM1_ESM.pdf]

Case Study Summaries – Additional File 1

This file present summaries of case studies explored by Together4RD in 2022, of examples of where groups or networks of rare disease stakeholders have collaborated/ do collaborate effectively with Industry. The first set of case studies focus primarily on **registries**:

ERK-REG

ERK-REG is the registry of the ERKNet ERN, for rare renal diseases. It was initiated in 2019 and acts as a single core registry for all rare renal diseases. The Registry collects data from the HCPs which are part of the ERN - this is a mix of epidemiological data concerning diagnostics, phenotypic and natural history data, and data to enable continuous monitoring of the diagnostic and therapeutic performance of HCPs (whilst also assessing guideline adherence). It can also be used for the rapid identification of patient cohorts for clinical trials. To-date, collaboration with Industry has included ERK-REG brokering contracts with sites that have patients eligible for clinical trials, and the provision of aggregate data on over 200 paediatric patients receiving a medicine off-label (which was used as supportive evidence for a Paediatric Investigation Plan). Over the first 3 years, 12,661 patients were enrolled, from 41 paediatric and 17 specialised adult units across 20 countries [44].

TREAT-NMD

This case study spans both the Registry and broader Clinical Research categories, as several different types of activities take place under the TREAT-NMD banner. TREAT-NMD was established back in 2007, via an FP6 grant, as a network to advance trial-readiness in all neuromuscular diseases. It has created a suite of tools and activities to achieve this goal, and in 2019 was 'spun out' of the University which coordinated it, as a legal entity. Key resources include cell and animal standard operating protocols (preclinical research); an advice service (TACT), global patient registries, ethical framework and care guidelines, and family guides, to help develop and extend translation research in the field. Many of these activities have involved Industry, and ethically-robust practices and codes have been developed to facilitate this. One key area of

Industry engagement concerns patient registries. TREAT-NMD links numerous registries (and developed core and expanded datasets to standardise data in these standalone registries) to facilitate the identification of specific patient groups and boost patient recruitment. It also coordinates global patient registries for several NMDs. The inter-connected registries provide a wealth of information and can be queried by academic sites (free) or by Companies (for a fee).

Sanofi Genzyme French Pompe Registry

This is an example of a national registry established by Industry. The registry was first qualified by French authorities in 2008, to collect prospective clinical, functional and biological data on all French patients with a Pompe disease diagnosis (whether treated or not). Sanofi Genzyme partners with a group of French hospitals, with contracts formed (and updated) with each hospital individually to provide the data. Funding comes from Sanofi to the research teams of the hospital, but is also provided through AFM (Association Française contre les Myopathies) and other French institutions.

The results of the Registry data collection are published annually [45] .

European Society for Blood and Marrow Transplantation (EBMT) Registry

The not-for-profit EBMT was founded in 1974 and is now dedicated to research, education and care for clinical bone marrow transplantation and cellular therapies. The Registry contains clinical patient data on diagnosis and disease, first-line treatments, haematopoietic stem cell transplant (HSCT) or cell-therapy-associated procedures, transplant type, donor type, stem cell source, complications, and outcome. EBMT provides data to its 5000 members (ranging from individual physicians and nurses to Centres) and is able to perform studies and assess epidemiological trends. Industry are long-time collaborators in the EMBT broadly. Companies can become partners of excellence, without voting rights. If they wish to participate in one of the EBMT scientific studies, using the Registry, they negotiate payment. Of particular note is the success in gaining EMA qualification (specifically of the cell therapy module): to-date, EBMT has made various agreements with Companies on a 1-2-1 basis to support their Post Authorisation Safety Studies,

based on secondary use of the Registry data. No direct access is provided to the Registry – rather, Companies approved as partners of excellence can access data collected in reports. In addition, Companies can contract for individual research projects, such as feasibility reports, surveys, conducting retrospective or prospective studies, etc. Where possible, only anonymous data is shared (pseudonymised data *can* be shared with explicit patient consent). The Registry produces over 100 publications per year [46] .

Further case studies were gathered, which are less concerned with registries but rather dedicated to optimising the **planning and delivery of clinical research more broadly**:

TREAT-NMD

More on the 'clinical research' side, the CTSR (care and trial site registry) was established to provide information on personnel facilities, patient populations, and prior experience with conducting clinical trials. Sponsors can use the resource to help select experienced trial sites. A key resource is the TREAT-NMD Advisory Committee for Therapeutics (TACT), which allows a better prioritisation of compounds to be taken from preclinical studies into clinical trials. For over a decade, this unique multidisciplinary enterprise has allowed academic groups and Companies to have their compound and clinical development plan evaluated by an international team of preclinical, clinical, regulatory, and industrial experts as well as patient representatives. Companies pay for this advice, and again the funds support the core activities of TACT. A high level of confidentiality is maintained.

Other interactions have included Industry funding training events, summer-schools and educational webinars (without playing a direct role in setting agendas). For many years, contracting was performed by Newcastle University, but recently TREAT-NMD Services Ltd launched as a not-for-profit legal entity.

Proof of Concept Club (POC Club)

The POC Club is a national (French) resource to promote innovative research and develop new treatments for rare diseases by offering coaching and guidance for researchers and clinicians and, crucially, connecting them with suitable Industry partners. It uses a business model based on a research valorisation tool. It was

set up in 2017 by the Foundation Maladies Rares, to bridge the gap between academia and industry in rare disease. Webinars allow academics to present 10 minute ‘elevator-pitches’ for projects they wish to conduct, and the Industry feasibility of the research proposals is assessed. 9 sessions have taken place so far, with 74 projects presented to POC Club. If a project is taken forward by a Company, partnership agreements can be signed between the key actors and a tech transfer office.

Innovative Therapies for Children with Cancer (ITTC) Consortium

The Consortium involves 63 Paediatric Oncology Departments in 18 European countries with expertise in conducting early phase trials in children and adolescents, and 25 European research laboratories. ITTC launched in 2003, long before the Paediatric Cancer ERN (ERN PaedCan). ITTC is a non-profit organisation established under French Law. The goal is to accelerate the introduction of new effective and safe therapies in the treatment of children and adolescents with cancer. The ITTC offers a wide range of collaborations with Industry, across the whole lifecycle of product development, from early portfolio evaluation to advice on generating phase 1 data, through to support with paediatric trial design and finally implementation. In this way, ITTC increases the likelihood of the most effective therapies entering development, optimising the chances of these medicines eventually becoming available to patients.

European Collaboration for Epilepsy Trials (ECET)

This is a relatively new collaboration, launched in 2021, between the ERN EPICARE and Epilepsy Alliance Europe. The goal is to provide advice and expertise to optimise the design and implementation of clinical trials in epilepsies. No reviews have yet taken place, but structures are being develop to contract with pharma companies and CROs [47].

ACCELERATE

The ACCELERATE Consortium was initiated in 2012, in the paediatric cancer field. The goal is to accelerate the process of developing and evaluating innovative therapies for children and young people with cancer. Pharma companies participate in the activities, which are essentially multistakeholder fora in which the

group analyses the state quo of research and development and identifies activities necessary to drive forwards the pace of progress. Industry is part of the Steering Committee and is part of projects designed to push the field forward (which include education and working groups on clinical research topics, but not specific trials). https://www.accelerate-platform.org/
