# Supplementary material for: Together4RD position statement on collaboration between European reference networks and industry
Source: Orphanet J Rare Dis. 2023 Sep 5;18:272. doi: 10.1186/s13023-023-02853-9 (PMC10478454; doi:10.1186/s13023-023-02853-9)
Supplement: Supplementary file 2 — Additional file 2. ‘Activities suitable for ERN and Industry Collaboration (Registries)’ (A table of data generated by a Together4RD Working Group to support creation of this Position Statement). [file 13023_2023_2853_MOESM2_ESM.pdf]

Additional File 2: Table of Activities suitable for ERN and Industry Collaboration - Registries

Types of collaboration involving Registries that should/could be pursued	Points to consider/ best practices identified by Together4RD Working Groups	Case Studies - examples of each type of collaboration in action
Using registry data to understand the natural history of a disease or identify unmet medical need	Longitudinal data collection can help to elucidate the natural history (NH) of a condition. Not enough is known about the NH of many rare diseases, which lack adequate registries to collect data (and often, for the rarest conditions, such registries need to operate at the global level). As registries are all about structured data, careful thought must be given to the initial data dictionary (including any associated mandatory or recommended datasets), as it needs to be sufficiently broad to detect *unknown* effects as well as monitoring known symptoms (i.e. without a robust starting knowledge of the NH, especially of complex multisystem conditions, it may be that a meaningful data item which *should* be monitored will not be recorded). Patient engagement in establishing registries to collect NH is therefore especially meaningful.<br><br>Related to this, registries hold the potential to illuminate unmet medical *need*; for instance, a registry dedicated to a rare lung or rare liver disease may capture other presentations or comorbidities that have not emerged in clinical trials, and which -in addition to elucidating natural history- therefore highlight unforeseen medical needs. It is important for Companies to know the full clinical picture. However, it is worth considering whether registries are the most appropriate sources of such knowledge - electronic health records (EHRs) possibly hold more potential here.	An example here is the Sanoffi French Pompe registry, which has been prospectively gathering clinical, functional and biological data of all French patients with a diagnosis of Pompe disease confirmed by enzymatic and/or molecular analysis, whether treated or not - untreated patients can help to reveal NH of the diseases.

Using registry data as real-world data to serve regulatory purposes	This is an often-cited goal for rare disease registries, but examples are quite challenging to find. One goal would be reducing the use of placebos in future trials by using registry data as a control arm. Such an activity is arguably more feasible and effective when data in registries is more standardised (and it is probably necessary to think less about ontologies, as has been the case with rare disease diagnostic platforms and project traditionally, and think increasingly of standards specifically relevant for clinical trial data, especially CDISC). There is a real need for regulatory buy-in for these sorts of uses, and there is still perhaps quite a poor understanding of what sort of data is acceptable to the regulatory bodies for particular types of activity. (The EMA has issued some guidance here, to which EJP RD partners provided input - https://www.ema.europa.eu/en/guideline-registry-based-studies-0)<br><br>A key point here is, to serve this kind of ambitious use, registries need to be collecting the right sort of data - data which will be of use to Sponsors and Regulators	ERK-Reg has been able to provide aggregate data on over 200 paediatric patients receiving a medicine on an off-label basis, to be used as supportive evidence for a Paediatric Investigation Plan. This data broadens the evidence base especially for safety, but also the efficacy of the drug, to inform the regulatory process. The EBMT registry has received a positive EMA qualification opinion, making its cellular therapy module a suitable data source for regulatory purposes. Both therefore offer insights to this kind of activity.
Using registry data to conduct post-marketing surveillance (see also a related activity below)	This is another type of activity which tends to be viewed as highly desirable, but apparently happens little in practice at present. A key consideration here is that patient-level data would be required for this. It has long been a goal of registries to replace the need for Industry to create drug-specific registries, but Companies frequently reply that existing registries are not capable of meeting strict regulatory criteria. Therefore, a real partnership between the registry creators/managers, Companies, and the EMA, would be required. In particular, tools would be required to ensure the quality of data in registries. The EMBT registry case study should be illuminating here.	EBMT has entered into various agreements with industry partners to support their EMA-mandated Post Authorisation Safety Studies (though even here, the studies are based on secondary use of EBMT registry data). The EBMT registry *does* include data quality checks that should promote consistency at the point of data entry, but there is no onsite Source Data Verification (SDV) or comprehensive remote SDV in terms of the entire registry as a whole; however, within the context of individual studies, additional quality checks can be

		performed (remote and/or onsite). The experiences of the few rare disease registries (EBMT and Cystic Fibrosis) which have received EMA qualification should be leveraged here, along with any guidance from the EMA Registry Taskforce. The TREAT-NMD Registries Platform is also an interesting example, as here, the goal is to enable multiple Companies to fund a common platform for PMS.
Collaborating on defining data sets or data dictionaries	It is important to consider the *purpose* of a registry – what must it be able to do? The data one needs to collect for a simple epidemiological study will be less than (and different to) data required for Post-Marketing-Surveillance. Several projects are looking strategically and technically at how to increase the interoperability and FAIRness of registries (along with other sources of rare disease patient data), to try to allow data to speak with other data from other relevant registries, to serve particular goals. These activities (e.g. the work on making the new ERN registries more FAIR under the EJP RD; the data tasks of conect4children which are exploring how registry data could support better clinical trials or function as RWD in the paediatric space; and disease-specific projects like Duchenne Data Project in the Netherlands)present a number of important best practices. **However, there has been less emphasis to-date on co-developing key resources like data dictionaries *with* Companies** (likely because the ERNs have not been able to do this to-date). Such activity, in the future, should include defining and implementing Patient Centred Outcome Measures within registries (aligning with work on PCOMs and PROMs under ERICA and the future RD Partnership, for instance)	The TREAT-NMD registry work is one example of where Companies *have* been involved in developing disease-relevant datasets and dictionaries for the global registries.
Use of registries to improve care	**I**t is very much in the interests of Companies to see the standard of care raised, which can happen when clinicians, researchers and patients use registry data to identify good practices and enshrine 'what works' into clinical practice	A good example here comes from the DMD field: registry data enabled a good understanding of NH but also showed what worked, in terms of

	guidelines or similar. Implementation of such guidance can create a more harmonised clinical ecosystem, which presumably then means a more equal baseline for patients with the same disease in different countries.	interventions – researchers could see that in countries where steroids were used, boys were ambulant for longer than in countries where they weren't provided routinely, and night-time ventilation improved health and wellbeing significantly, etc. Those observations then made their way into international diagnosis and management guidelines, which are an important tool for standardising the level of care.
Use of registries to identify the best clinically-performing sites	Companies value knowledge about HCP/site expertise and outcomes. By benchmarking centres, companies can gain information of respective HCP outcomes, life expectancy etc. Registries can thus yield valuable information on regional and national performance and assist with decisions on which sites to contract with for clinical trials, as well as potentially supporting decisions on where to concentrate ATMPs provision.	The ERK-REG registry provides the ability to benchmark in this way
Using registry data to do feasibility assessments and trial planning	This is linked to the previous activity, but goes a step further.	A good practice noted in the ERK-REG case study is the brokering of Sponsor contacts with sites that have patients eligible for particular clinical trials: the registry allows Companies to assess the feasibility of their studies. Another good example comes from the TREAT-NMD registries, which use a global network of autonomous registries (most using core and extended datasets to promote more harmonised and interoperable data). A system of checks and balances is in place to ensure Companies can make a request to an oversight committee made up of curators of national

		registries for conditions like SMA and DMD. If this TGDOC, as it is called, approves the request, the data is collected from the registries (in aggregate form) and Companies can see how many patients they would likely be able to recruit in particular countries, how many patients meet particular inclusion criteria etc. This is very valuable in terms of letting Industry plan whether a trial is feasible or not and gives insight on how to structure it. The Companies pay for this aggregate data and the funds go back into the TREAT-NMD system, supporting the curators of the registries to meet and network, for instance. The EBMT also provides data for Companies, based on individual requests. Such research projects include (feasibility) reports, surveys, support for statistical analyses, performing retrospective or prospective studies (depending on the informed consent – specific projects sometimes require new informed consent forms, different to that requested when originally inputting data.)
Industry *funding* registries or registry platforms	This activity may involve some or all of the activities specified above, but goes a step beyond, in one key way - here, Industry contributes resources to the setting-up, maintenance or expansion of a registry/registry platform. There are multiple benefits here, including the ability to avoid the creation of drug-specific registries. It would probably be necessary to think of a collaborative funding approach here, with modules for specific conditions. As yet, no examples were forthcoming from the Together4RD stakeholders, so this would very much be an aspirational future goal for the ERN ecosystem to explore.	
