# Supplementary material for: Together4RD position statement on collaboration between European reference networks and industry
Source: Orphanet J Rare Dis. 2023 Sep 5;18:272. doi: 10.1186/s13023-023-02853-9 (PMC10478454; doi:10.1186/s13023-023-02853-9)
Supplement: Supplementary file 3 — Additional file 3. ‘Activities suitable for ERN and Industry Collaboration (Clinical Research)’ (A table of data generated by a Together4RD Working Group to support creation of this Position Statement). [file 13023_2023_2853_MOESM3_ESM.pdf]

Additional File 3 - Activities suitable for ERN and Industry Collaboration (Clinical Research)

Types of collaboration involving ‘clinical research’ which should/could be pursued	Points to consider/ best practices identified by Together4RD Working Groups
Broad (all-ERN) multistakeholder forums to build mutual awareness of achievements and open a dialogue	Given the lack of opportunities to-date for ERNs and Companies to enter into dialogue openly, it might make sense to create a dedicated once-a-year event for ERNs and Industry, for the latter to learn more about what ERNs are really doing and see where their strengths lie. This would be a relatively simple but important ‘catch-all’ activity, to help shape more specific collaborations. This is based somewhat on the idea of the Accelerate example, and also on the EURORDIS RoundTable of Companies, but in this option would be envisaged as a single forum for all Networks and all interested Companies to attend. It may be that such a meeting could be part of the EC-organised ERN conference (assuming these recommence, post-covid), or else could be envisaged as a standalone event. Perhaps individual ERN meetings with Industry could branch-off after the plenary.
Disease-specific (or area specific) multistakeholder forums to advance trial-readiness and prioritise collaborative activities	This is a similar activity to the previous, but here fora would be ERN-specific. Multistakeholder groups/fora, organised at more disease-area-specific levels, could be very beneficial to accelerate the pace of trial-readiness and maturation: a good example here is the Accelerate initiative, where all stakeholder, including Industry, gather to discuss the state of the art and identify strategic needs and gaps in their disease area/intervention. Accelerate organise such events for paediatric cancers, but specific fora could be established under the aegis of ERNs, perhaps funded by companies, with the programme created by academics and patients. Forums like these could address some of priorities this WG identified, in terms of ERN: Industry interactions, such as what patient-centred trials in that area look like, agreeing relevant endpoints for studies in X and Y diseases, etc.

A 'match-making' forum for researchers to pitch their ideas to Companies and bid for funding support	The case study of the French POC (Proof of Concept) is a good model for this sort of activity. There are certain requirements, if the POC would be replicated in other countries, or indeed established as a vast all-ERN opportunity. This would differ from the activities above, as here, specific research proposals from academics would be presented and assessed. If expanded to the ERNs, it is difficult to see how this would work on a national level; in France, the presence of French Tech Transfer Offices has been crucial. Perhaps a pan-European entity such as EATRIS or other similar body could play such a role, if POC events were organised along ERN lines. The role of the Foundation Maladies Rare here has been critical in the French POC example, as a 'Neutral and trusted third party' to initiate and facilitate the partnerships (of 75 presentations to-date, 56 connections have been made.)
Enabling assessments of clinical trial feasibility and/or finding patients for clinical trials	A number of activities can be identified which collectively help to de-risk clinical research in rare diseases for Industry. Registries can play a key role in this as (anonymised, aggregate) data can be provided to Companies to help them assess the feasibility of a study in a given condition, with particular inclusion criteria, in particular countries or regions. e.g. the TREAT-NMD case study shows us how registries have been used to inform Companies about the number of patients in particular countries or regions with a particular type of Duchenne Muscular Dystrophy, for instance, within a certain age range, who meet particular inclusion criteria (e.g. are still ambulant, have not taken steroids etc). The aggregate data provided by the national registries associated to TREAT-NMD can then be used to help that Company plan its trials effectively. See further the 'Registries' sub-group table. Another useful asset here is TREAT-NMD's CTSR (Care and Trial Site Registry), which is a registry not of patients but of sites, providing information on those sites, the cohorts they can provide, etc. Some ERNs have developed (and others may be developing) registries able to support with finding patients for trials - indeed, the ERK-Reg case study can perform such a role.<br><br>Naturally, any activity aimed at indicating to Companies how many patients they might be able to recruit for research and where they are based needs to be kept separate from actual recruitment efforts (but again, the case studies gathered by Together4RD ensure this a matter of good practice)
Providing expert, tailored and confidential advice to companies for optimised therapy development	Several case studies demonstrate the importance of this function, which, when provided within disease-specific fora, seems to have a major added-value beyond the sorts of early advice offered via Regulatory bodies alone, for instance. For instance, ITTC (Innovative Therapies for Children with Cancer) assesses the relevance of mechanisms of action for experimental paediatric oncology medicines and – if there is potential in the therapy – provides advice to a company on a 1-2-1 basis, ranging from early portfolio evaluation (preclinical) through to support for trial implementation. Having ITTC established as a non-profit legal entity (under French law) has facilitated this service.<br><br>The ACT (Advisory Committee for Therapeutics) model, which originated from the TREAT-NMD case study, was also presented as a good (and very replicable) model here. Work is ongoing under the EJP RD to try to take this model, used for over a decade in the neuromuscular field and apply it

	(with any necessary adaptations) to other RD areas, strategically overseen by ERNs wherever possible. Several fields have expressed interest. However, some form of seed funding is really required to do this well, until the model is established and becomes self-sustaining. Companies could foreseeably look at precompetitive funding of some kind (or if they know they will wish to use the services of an ACT in a given area in the near future, they might consider funding the initial costs.) For many years, the Neuromuscular ACT was run from a single University, which oversaw the contracting etc and used fees from Companies (on a sliding scale, depending on size of the Company) to pay for costs of the panel review meetings. In the absence of a new legal entity, this model could be replicated in ERNs by channelling contracting through a single HCP playing a leading role in the ACT for that ERN. The ECET (European Collaboration for Epilepsy Trials) case study is also starting to provide a trial advisory service in the epilepsy field.It is of course essential that such expert advice services maintain confidentiality for the Companies seeking them – the resources names above have developed templates and CDAs (indeed full toolkits, in some cases) which could be used here.
Creating/Improving biobanks	Some communities have their own disease-related biobanks. Other samples are part of very large biobanks and networks of biobanks e.g. EuroBioBank. Projects like RD-Connect embraced the EuroBioBank network and created a biobank and registry *finder.* BBMRI also maintains a biobank catalogue. It may be, however, that many fields are not using biobanks effectively and would benefit from support to do so. The status quo of rare disease biobanks is being addressed by the ERICA project - with greater understanding of the needs though, there *could* potentially be scope for cross-company Industry support of foundational biobanks for particular disease areas.
Diagnose patients for clinical research through EHRs from ERN centres (HCPs and 'affiliated' centres)	Together4RD has not yet received case studies of this happening; indeed, it is likely that this falls into the category of 'new activity which would be possible between ERNs, specifically, and Industry'. The fact that each ERN connects HCPs across the EU and EEA countries should, in theory, make it more feasible for electronic health record (EHRs) to be federated somehow, to enable the diagnosis of patients not currently diagnosed and enrolled in registries etc. There would be potential for AI approaches to be incorporated here. However, the scope of such activity would require careful consideration: if patients are coming to the attention of ERN HCPs, is it likely that they will remain undiagnosed (and if they are, would AI algorithms be able to solve these cases, or would referral to Solve RD or similar not be more promising?). To really optimise a diagnostic yield of previously undiagnosed patients, perhaps one would need to access EHRs in more general hospitals, rather than specialist clinics, or even in primary care settings. (N.B the Screen4Care IMI 2 project is exploring routes to early diagnosis of people with a RD, through Newborn Screening but also other routes – their work should be illuminating here, perhaps).Therefore, perhaps the added-value here would be less about diagnosing patients who do not have a diagnosis and rather finding patients with particular phenotypes, e.g. stratifying cohorts.

Defining strategic priorities for clinical research and drug development based on unmet medical need	This sort of activity can probably be achieved via the strategic forums (along the lines of what Accelerate does), outlined above. Furthermore, the future European RD Partnership should, via its anticipated Clinical Research Network(s), define priorities and dedicate particular efforts to drug development for RD with major unmet needs
