# Supplementary material for: Together4RD position statement on collaboration between European reference networks and industry
Source: Orphanet J Rare Dis. 2023 Sep 5;18:272. doi: 10.1186/s13023-023-02853-9 (PMC10478454; doi:10.1186/s13023-023-02853-9)
Supplement: Supplementary file 4 — Additional file 4. ‘Overview of other initiatives complementing the work of Together4RD’ (Summary illustrating how the mission of Together4RD fits into a broader ecosystem of projects and initiatives working towards a more collaborative ERN-Industry ecosystem). [file 13023_2023_2853_MOESM4_ESM.pdf]

Additional File 4 – Overview of other initiatives complementing the work of Together4RD

Entity	How does/will it complement the work of Together4RD?
BoMs Working group on legal and ethical issues (LES)	This WG is creating key documents referenced in the BoMS Statement of 2019, specifically a conflict-of-interest (CoI) policy and CoI form. It is looking at possible governance structures for ERN-Industry interactions. The case studies and desk-research of Together4RD should enrich this work, and the Together4RD Pilots should be developed and delivered through close dialogue with the BoMS broadly, and this WG in particular.
European Rare Disease Research Coordination and Support Action consortium (ERICA)	A 4-year project specifically to advance ERN research, which is developing resources to improve various aspects of rare disease research (e.g. developing more effective data collection strategies, optimising patient involvement etc.).The outputs of the data WP will likely be particularly relevant for Together4RD; for instance, as ERICA is assessing the status quo of biobanks, gaps identified here could support the development of targeted projects or pilots between ERNs and Industry
European Joint Programme on Rare Diseases (EJP RD)	EJP RD (2019-2023) has created important resources (especially relating to data) which should be considered by Together4RD pilots. Industry is partnering with researchers in key public private partnerships (though not with ERNs). Translational support services to link researchers and Industry are also available
conect4children (c4c)	c4c is an IMI2 project (2018-2024) establishing a European network and streamlined ecosystem for clinical trials in paediatric diseases. As so many paediatric diseases are also rare diseases, c4c's processes and tools to support better, more efficient and more successful clinical trials in children and young people also address broader rare disease needs. As an IMI project, c4c is naturally supporting collaboration between public and private sectors. c4c is expected to evolve into a legal entity by the time the IMI2 grant concludes. Considerations of how ERNs and Industry can work together should therefore factor in how the c4c landscape (centred on new national hubs, connected via a single point of contact for any Company seeking to conduct a paediatric trial in Europe) may be utilised, when paediatric patients are involved.
European Partnership on Rare Diseases (RDP)	Assuming Industry is able to engage as a full partner, the RDP (expected to start around mid-2024) should offer opportunities for Industry and researchers to collaborate in the pre-competitive space, particularly through projects under the heading of a European 'Clinical Research Network'. There could foreseeably be opportunities for more ERN-Industry pilots of the kind identified in Together4RD to be delivered through the RDP.

European Health Data Space (EHDS)	Some of the planned pilot activities for EHDS will be relevant to the mission of Together4RD (although timelines are uncertain). For instance, work will likely take place to help ERNs to establish minimum data sets, and EU support is expected to further evolve registries and increase use of data from registries for research and policymaking.
DARWIN EU	DARWIN EU is establishing a network of data, expertise and services to support better decision-making. Some of the activities identified for ERN and Industry collaboration are very relevant here, and Together4RD pilots should follow DARWIN good practices closely (and vice versa)
Screen4Care	An IMI2 initiative, looking at earlier diagnostics for people with a rare disease, both via expanded newborn screening but also use of AI to identify patients with early onset disease through EHRs – this latter goal, in particular, could be synergistic with Together4RD.
Moonshot for Rare Diseases	EFPIA and EURORDIS have proposed a Moonshot for RD, with a global dimension, to focus particularly on neglected diseases which have no dedicated approved treatments. The sorts of activities Together4RD has been exploring and distilling (sections 3.3-4) and the pilot proposals gathered, should be illuminating for the Moonshot.
